# Purification of Immature Neuronal Cells from Neural Stem Cell Progeny

**DOI:** 10.1371/journal.pone.0020941

**Published:** 2011-06-03

**Authors:** Hassan Azari, Geoffrey W. Osborne, Takahiro Yasuda, Mohammad G. Golmohammadi, Maryam Rahman, Loic P. Deleyrolle, Ebrahim Esfandiari, David J. Adams, Bjorn Scheffler, Dennis A. Steindler, Brent A. Reynolds

**Affiliations:** 1 Queensland Brain Institute, The University of Queensland, Brisbane, Australia; 2 Laboratory for Stem Cell Research, Department of Anatomical Sciences, Shiraz University of Medical Sciences, Shiraz, Iran; 3 Department of Neurosurgery, McKnight Brain Institute, The University of Florida, Gainesville, Florida, United States of America; 4 Health Innovations Research Institute, RMIT University, Bundoora, Victoria, Australia; 5 Department of Anatomical Sciences, Ardebil University of Medical Sciences, Ardebil, Iran; 6 Department of Anatomical Sciences, Isfahan University of Medical Sciences, Isfahan, Iran; 7 Institute of Reconstructive Neurobiology, University of Bonn, Bonn, Germany; University of Milan-Bicocca, Italy

## Abstract

Large-scale proliferation and multi-lineage differentiation capabilities make neural stem cells (NSCs) a promising renewable source of cells for therapeutic applications. However, the practical application for neuronal cell replacement is limited by heterogeneity of NSC progeny, relatively low yield of neurons, predominance of astrocytes, poor survival of donor cells following transplantation and the potential for uncontrolled proliferation of precursor cells. To address these impediments, we have developed a method for the generation of highly enriched immature neurons from murine NSC progeny. Adaptation of the standard differentiation procedure in concert with flow cytometry selection, using scattered light and positive fluorescent light selection based on cell surface antibody binding, provided a near pure (97%) immature neuron population. Using the purified neurons, we screened a panel of growth factors and found that bone morphogenetic protein-4 (BMP-4) demonstrated a strong survival effect on the cells *in vitro*, and enhanced their functional maturity. This effect was maintained following transplantation into the adult mouse striatum where we observed a 2-fold increase in the survival of the implanted cells and a 3-fold increase in NeuN expression. Additionally, based on the neural-colony forming cell assay (N-CFCA), we noted a 64 fold reduction of the *bona fide* NSC frequency in neuronal cell population and that implanted donor cells showed no signs of excessive or uncontrolled proliferation. The ability to provide defined neural cell populations from renewable sources such as NSC may find application for cell replacement therapies in the central nervous system.

## Introduction

Cell-based therapy in neurological diseases is an attractive option, but presents a difficult challenge, given the diversity of central nervous system (CNS) cell types, the complex and precise interactions amongst them and the availability of appropriate cellular sources. Sources for cell transplantation in the nervous system includes fetal neural tissues [1], [2], [3], embryonic stem (ES) cells [4], [5], induced pluripotent stem (IPS) cells [6], neural stem cells (NSCs) [7], [8], non-neural somatic stem cells [9], [10], [11], [12] or even direct conversion of non-neural cells into neurons [13]. Each of these cell types have the potential to replace cells lost to injury or disease [14] or to modulate brain or spinal cord function [15]; with each having their own advantages and disadvantages. Among the available options, NSCs are a promising choice as they retain the ability to generate a large number of cells from a relatively small amount of starting tissue and express the capacity for multi-lineage differentiation [16], [17]. However, NSC progeny are a heterogeneous cell population that exhibit poor survival [18] and largely differentiate into glia following implantation into the mature CNS [8]. In addition, a small population of the NSC progeny may retain a substantial proliferative potential [19], [20]. These caveats are further compounded by the poorly defined composition of cells within a multi-lineage NSC culture and the need for well characterized, highly purified cell phenotypes so as to reduce variability in pre-clinical and clinical investigations.

To overcome these problems it is desirable to establish standard reproducible methodologies to generate highly enriched or relatively pure populations of cells. These cells can also be used for screening assays to uncover agents or niche-related conditions that enhance their survival, differentiation, neurite outgrowth and integration into the pre- existing circuitry of the adult CNS. With these aims in mind, and using cultured NSCs as a starting source of cells, here we show that using the distinct morphological characteristics of glial and neuronal cell populations, derived from differentiating NSC progeny, an enriched population of immature neurons can be isolated based solely on cell size and internal complexity (i.e. forward and side scatter properties; FSC and SSC). This enriched neuronal population contains a significant reduction in contaminating stem and progenitor cells, as evidenced by the *in vitro* neurosphere and neural colony forming cell (N-CFC) assays. Further purification (up to 97%) of the neuronal population can be achieved by excluding O4 and A2B5-immunoreactive (IR) cells (negative selection) or by positively selecting the PSA-NCAM-IR cells within the FSC^low^SSC^low^ cell population. Screening a small panel of growth factors, we identified BMP-4 as a factor supporting the survival and maturation of the purified immature neuronal cells *in vitro* and following transplantation. Importantly, implanted cells retained their neuronal phenotype and showed no signs of excessive proliferative ability.

Development of similar methodologies for purifying astrocytes and oligodendrocytes will provide the opportunity to deliver defined populations of cells into the CNS with the intent of enhancing donor integration and ultimately modifying host physiology.

## Methods

### Ethics Statements

Animals used in this study including adult male non obese diabetic-severe combined immunodeficient (NOD-SCID) and C57-BL6 wild type (Animal Resource Center, Nedlands, WA) and doublecortin-GFP and Tau-GFP mice (from Dr. Helen Cooper Laboratory, The University of Queensland, Australia) were housed with unlimited access to food and water. The University of Queensland Animal Ethics Committee approved all experimental protocols in this study (Approval ID#QBI/173/06/QBI).

### Neural stem cell isolation, expansion and differentiation

Neural stem and progenitor cells from the ganglionic eminences of E_14_ brains of C57-BL_6_ background (wild-type, doublecortin-GFP and tau-GFP) mice were isolated and expanded using the neurosphere assay [16], [21] . Primary neurospheres were passaged after 5–7 days using 0.05% trypsin-EDTA (Gibco, USA) and plated at a density of 5×10^4^ cells /ml in mouse NeuroCult basal NSC Medium plus mouse Neurocult NSC Proliferation Supplements (Stem Cells Technologies, Vancouver, Canada) supplemented with 20 ng/ml EGF (BD Biosciences, San Jose, CA) in NUNC tissue culture flasks in humidified 37°C incubator with 5% CO_2_. To differentiate neural stem cells progeny, we employed the method used by Scheffler et al [22] with some modifications. Briefly, dissociated neurospheres from passage one to three were plated at a high density (3×10^5^ cells/ml) in mouse NSC medium containing 5% fetal calf serum (FCS) (Gibco), 20 ng/ml EGF, 10 ng/ml bFGF (Roche, Basel, Switzerland) and 2 µg/ml heparin (Sigma) for 3–4 days. Subsequently, the medium was switched to the same medium with 5% FCS but without any growth factors. At this stage neuronal progenitors start to divide repetitively and generate colonies of immature neurons (neuroblast cells) within 4 days. Hence we call this assay the NeuroBlast Assay (NBA) so as to distinguish it from conventional differentiation methods.

### Cell preparation and flow cytometry

Four days after switching to growth factor free medium, neuroblast assay cultures were washed with phosphate-buffered saline (PBS) and treated with pre-warmed trypsin-EDTA for 1–2 minutes in a 37°C incubator. An equal volume of trypsin inhibitor (prepared as 0.014% w/v trypsin inhibitor (type I-S from soybean; Sigma- Aldrich, St. Louis, MO) in HEPES-buffered minimum essential medium (HEM), which consisted of minimum essential medium (Invitrogen) and 16 mM HEPES (Sigma-Aldrich) was used to quench trypsin activity. The cell suspension was centrifuged at 700 rpm for 5 minutes. After resuspending in 2–3 ml of NSC medium, cells were passed through a 40 µm cell strainer (BD, Falcon, USA) to separate non-dissociated clumps. Propidium Iodide (PI, Sigma, 500 µg/ml in PBS) was added at a concentration of 1 µl/ ml of cell suspension to exclude dead cells when analyzed by flow cytometry. To exclude contaminating cells, and to have further neuronal enrichment, antibodies against the oligodendrocyte maker O_4_ (Chemicon, monoclonal anti-IgM, 1∶300) and bipotential glial cells marker A_2_B_5_ (Chemicon, monoclonal anti-IgM, 1∶300) were added to the cell suspension for 30 mins at room temperature. For the direct isolation of immature neuronal cells, anti-PSA-NCAM antibody (Chemicon, monoclonal anti- IgM, 1∶300) was added to the cell suspension, and incubated for 30 min at room temperature. After washing twice with basal medium, cells were resuspended in fresh medium containing secondary antibody (Chemicon, goat anti-mouse IGM-Alexafluor 633, 1∶700) for 30 min at room temperature in the dark. Alternatively, when sorting PSA-NCAM IR cells from the O4/A_2_B_5_ double excluded cell population, Phycoerythrin (PE) conjugated anti-PSA-NCAM antibody (Miltenyi biotec, USA) was used at 10 µl/ 1×10^6^ cells in 100 µl of NSC medium for 10 mins in the dark at 2–8°C. After washing twice in medium, cells were resuspended in 2–3 ml of medium with PI, analyzed and sorted by a FACS Vantage SE Diva (BD Biosciences, CA, USA), running phosphate buffered saline (PBS) as the sheath fluid at 28 PSI through a 90 µm nozzle. Differential pressure on the system was adjusted such that sort trigger rates did not exceed 800 events/second. Laser excitations for fluorescence measurements were 200 mW at 488 nm or 35 mW at 633 nm. Resulting data were gated on bivariate displays, initially on forward and side scatter pulse area, to exclude debris and unwanted cells, and then on side scatter pulse width, versus side scatter pulse height to exclude doublets or cell clumps. Subsequent gates were set to exclude dead cells (PI positive cells) and select the cells, which represented the different cell populations of interest and/or showed staining above or below background. These sorted cells were collected in 15 ml BD Falcon tubes containing NSC medium. After spinning and performing a cell count, cells were plated at 1×10^4^ cells/well in poly-l-ornithine (Sigma, USA) coated 96 well plates containing mouse NSC medium alone or the medium supplemented individually with growth factors (fetal calf serum (FCS) at 2% and 10%, brain derived nerve growth factor (BDNF, human recombinant, R&D system, USA) at 100 ng/ml, ciliary neurotrophic factor (CNTF, human recombinant, R&D system, USA) at 20 ng/ml, growth hormone (GH, human recombinant, R&D system, USA) at 100 ng/ml, noggin (mouse recombinant, R&D system, USA) at 200 ng/ml and BMP-4 (human recombinant, R&D system, USA ) at 20 ng/ml).

### Apoptosis assay

To further evaluate the survival effect of BMP4, sorted immature neuronal cells were plated at a density of 4×10^6^ cells in 5 ml of NSC medium supplemented with or without 20 ng/ml of BMP-4 in T25 flasks. One day after sorting, cells were detached from the culture flasks using trypsin-EDTA and either used for Annexin-V assay (Molecular Probes) or fixed using cold 4% paraformaldehyde (PFA, Sigma Aldrich, USA) and immunostained for rabbit anti-active Caspase-3 (BD Biosciences, 1∶500) and β III-tubulin (Promega, 1∶2000) to evaluate the rate apoptotic cell death. Using BD LSR-II flow cytometer, the percentage of total PI^+^ cells (dead or dying cells) and Annexin-V IR cells (apoptotic cells) out of total live cells and the percentage of total active Caspase-3 IR cells (apoptotic cells) out of total β III-tubulin IR were calculated for both control and BMP4 treated cells.

### Immunofluorescence and microscopy

At different time point after sort, cultured cells were fixed for 20 min using cold 4% PFA and washed with PBS. The primary antibody solution prepared at appropriate concentration in PBST (PBS +0.1% Triton-X) with 10% normal goat serum (NGS, Sigma-Aldrich) was added to each well of the 96 well plates and incubated for 1 hour at room temperature, or overnight at 4°C. Primary antibodies used in this study included rabbit polyclonal antibodies against GFAP (DakoCytomation, 1∶500), DARPP **(**dopamine- and adenosine 3, 5-monophos- phate-regulated phosphoprotein) -32 (Chemicon, 1∶500), TH (Abcam, 1∶500), GAD 65/67 (Chemicon, 1∶500), and ChAT (Chemicon, 1∶100), and mouse monoclonal antibodies against β III-tubulin (Promega, 1: 2000), O_4_ (Chemicon, 1∶300), A_2_B_5_ (Chemicon, 1∶300), Map2 (Chemicon, 1∶300), Nestin (Chemicon, 1∶200), PSA-NCAM (Chemicon, 1∶300), and NeuN (Chemicon, 1∶150). Then, the primary antibody was gently removed from the wells and the wells were washed with PBS. Secondary antibodies were prepared at appropriate concentrations in PBST (PBS +0.1% Triton-X) with 10% NGS, and were added at 50 µl/well and the plate was incubated for 45 min at room temperature in the dark. Secondary antibodies used in this study included Alexa fluor 568, Alexa fluor 488, Alexa fluor 633 conjugated goat anti mouse or anti rabbit antibodies (all from Molecular Probes, 1∶700). 4′,-6′ diamidino-2-phenylindole (DAPI) (Molecular Probes, 1∶1000) was also added to the secondary antibody solution to label nuclei. Representative pictures of each well (10–15 fields/ well) were taken using a fluorescent microscope (Olympus IX-70) equipped with Canon EOS digital camera and cell counts were performed and calculated as a percentage of total cells counted, or mean absolute number/ field of view.

### Determining the frequency of neural stem/progenitor cells in sorted cells

To evaluate sphere forming frequency, sorted cells from P1, P2 and the total population (P4) were counted and plated in 250 µl/well of NSC medium supplemented with 20 ng/ml EGF in 96 well plates (NUNC) at the density of 1×10^4^, 4×10^3^, and 5×10^3^ cells/well respectively. Neurospheres greater than 50 µm were counted 7 days after plating and expressed as a percentage of total cells plated.

To evaluate bona fide neural stem cells frequency, 1×10^4^ cells/dish from P1, 4×10^3^ cells/dish from P2, and 5×10^3^ cells/dish from the total population were plated in 35 mm cell culture dish (NUNC) in 1.5 ml/ dish of serum-free neural colony forming cell assay (N-CFCA) medium containing supplements as described in the Neural Colony Forming Cell Assay kit (Stem Cell Technologies, Vancouver, Canada). 20 ng/ml EGF was used as a mitogen. The culture was fed every 7 days with 60 µl / dish of the NSC medium with EGF. Twenty-one days after plating, colonies were counted, sized and the number of big colonies (greater than 2 mm) was expressed as a percentage of total cells plated representing the frequency of the bona fide neural stem cells [23], [24].

### Immunophenotyping and electrophysiological propeties of sorted neuronal cells

Cells from the P1 population were plated at a density of 1×10^5^ cells/well on poly-l-ornithine (Sigma, USA) coated coverslips in mouse NSC medium supplemented with 20 ng/ml BMP-4_,_ astrocyte conditioned medium, and astrocyte conditioned medium supplemented with 20 ng/ml of BMP-4. To prepare astrocyte conditioned medium, NSC medium supplemented with 5% FCS was added to a sister flask of differentiating NSC culture 12 hours before sort. Half of the medium was refreshed with the same medium in each condition at 4 and 8 days after sorting and then every 3 days up to two weeks. Addition of BMP-4 was discontinued from the day 10 after plating. Cells were either fixed with PFA at 7 days post sort and or have been used for electrophysiology study at 2 weeks post sort. Different antibodies including anti-Map-2, anti-NeuN, anti-GAD65/67, anti ChAT, anti-TH, and anti-DARPP-32 were used to identify the phenotype of sorted neurons. After taking pictures, cells were quantified and expressed as a percentage of total neuronal cells counted.

Electrical membrane properties were recorded using the whole-cell patch clamp recording technique. Patch electrodes were pulled from borosilicate glass capillaries with resistances of 1.5–3.0 MΩ when filled with an intracellular solution containing (in mM): 5 NaCl, 145 KCl, and 10 HEPES-NaOH, pH 7.2. Cells were maintained in physiological saline solution of the following composition (in mM): 140 NaCl, 3 KCl, 2 CaCl_2_, 1 MgCl_2_, 10 glucose, and 10 HEPES-NaOH, pH 7.4. Experiments were performed at 20–23°C. Data were acquired at 5–10 kHz using an Axopatch 200A patch-clamp amplifier and a Digidata 1320 interface (Molecular Devices, Sunnyvale, CA) linked to a personal computer equipped with pCLAMP 9.0 software and filtered at 2–5 kHz. The resting membrane potential (RMP) and input resistance (R_in_) was determined from a membrane potential without current injection and from the steady-state voltage response to a hyperpolarizing current pulse of 10–50 pA, respectively. The membrane time constant (τ_m_) was determined from a single exponential fitted to the voltage response obtained with the hyperpolarizing current step, and cell membrane capacitance (C_m_) was calculated from R_in_ and τ_m_. Depolarizing current pulses of varying amplitude were applied to determine if action potentials (APs) could be evoked. In the case that no evoked APs were observed at the RMP, cells were held at −60 mV and the occurrence of evoked APs was assessed. Data were analyzed using Clampfit 9.2 and Prism 4.0 (GraphPad, San Diego, CA).

### Transplantation

Adult (6–7-weeks of age) male NOD-SCID and C57-Bl6 wild type mice were anesthetized using 3.5 µl/gr of a five times diluted mixture of K.X.A (Ketamine (100 mg/ml, 1.5 ml), Xylazine (20 mg/ml, 1.5 ml) and Ace promazine (10 mg/ml, 0.5 ml). Immature neurons were prepared as previously described, solely based on FSC and SSC from differentiating neural stem cells isolated from tau-GFP E_14_ mice of C57/Bl6 background. 2 µl of cell suspension containing 1×10^5^ cells in NSC medium alone (C57/BL6, n = 4 and NOD-SCID, n = 4) or supplemented with 40 ng/ml BMP_4_ (C57/BL6, n = 4) were injected through a burr hole using a 5 µl Hamilton syringe (Hamilton Co. USA) over 5 minutes into the right striatum at the following coordinate: A/P +0.5 mm, Lat −2 mm and D/V −4 mm. Animals were deeply anesthetized at 4 weeks post transplantation with sodium pentobarbitone (260 mg/kg), then transcardially perfused with 0.9% saline followed by cold 4.0% paraformaldehyde in 0.1 M phosphate buffer (pH 7.4). Brains were post-fixed in 4% PFA overnight at 4°C, washed with PBS and cryopreserved in 30% sucrose in PBS for 24–48 hours. Then, the brains were blocked in optimal cutting temperature (OCT) on liquid nitrogen and stored in −80°C freezer until needed.

Brains were cut in a systematic random fashion at 14 µm thickness with a Leica CM3050 cryostat. Sections were taken as 4 series (collecting every 4th section on each slide) from the beginning to the end of the striatum and mounted on superfrost plus slides, dried at room temperature and stored at −20°C until needed.

### Microscopy and cell quantification

To evaluate the proliferative potential of transplanted cells, after performing heat antigen retrieval (retrieval solution, Dako), immuno-fluorescence staining was performed on a series of sections in 0.3% PBST solution with 10% normal goat serum (NGS) containing primary antibodies against GFP (Chicken anti GFP; abcam, 1/500) and cell proliferative markers such as Ki-67 (Rabbit anti Ki-67; Lieca, 1/500) and MCM2 (Rabbit anti MCM2; invitrogen, 1/500). Sections were left in primary antibody solution for 36 hours at 4°C. Then, primary antibody was gently removed and the sections were washed with PBS. Secondary antibodies were prepared at appropriate concentrations in PBST (PBS +0.3% Triton-X) with 10% NGS, and left on sections for 1 hour at room temperature in the dark. After washing, sections were coverslipped, using Vectashield mounting medium containing DAPI (Vector Laboratories, CA, USA). Representative images were taken for each animal using Olympus DSU-IX81confocal microscope and the percentage of Ki-67 and MCM2 positive cells out of total GFP/DAPI-IR counted cells were calculated.

To estimate the number of cells survived four weeks after transplantation, a series of sections (systematic random sections representing the total transplant region) from each animal was immunostained for NeuN (Chemicon, 1∶150) and GFP (Chicken anti GFP, AVES lab Inc, 1/500) as previously described. Images from the entire graft region of each stained section were captured on a Canon EOS digital camera using an Olympus Axiophot upright fluorescence microscope. Images were merged together and the brightness and contrast were adjusted using Adobe Photoshop CS4. The total number of DAPI stained GFP positive cells was counted for each section and pooled for each series (∑Q^_^) then multiplied by the reciprocal of section sampling fraction (1/SSF) to have an estimate of total cells in each animal (E (N)) (Fractionator method; E (N) = ∑Q^_^×1/SSF×1/ASF×1/TSF)[25]. Area sampling fraction (1/ASF) and thickness sampling fraction (1/TSF) are equal to one as we counted cells in all graft area through the entire section thickness. To avoid overestimation, only GFP-IR cells with clear DAPI positive nuclei were counted. In the same sections the number of GFP-NeuN positive cells was quantified and expressed as a percentage of total GFP positive cells. The NeuN-GFP double labeling was confirmed using a confocal microscope (Zeiss Meta 510).

### Statistical analysis

One-way analysis of variance (ANOVA) or Students *t*-tests were used to analyze data as appropriate (Prism 4, Graphpad Software Inc. USA). Significant ANOVA values were followed by post hoc comparisons of individual means where applicable. All values are expressed as mean±standard error of the mean. The level of significance for all comparisons was p<0.05.

## Results

### Isolation of immature neuronal cells based on physical characteristics

**Figure 1:**
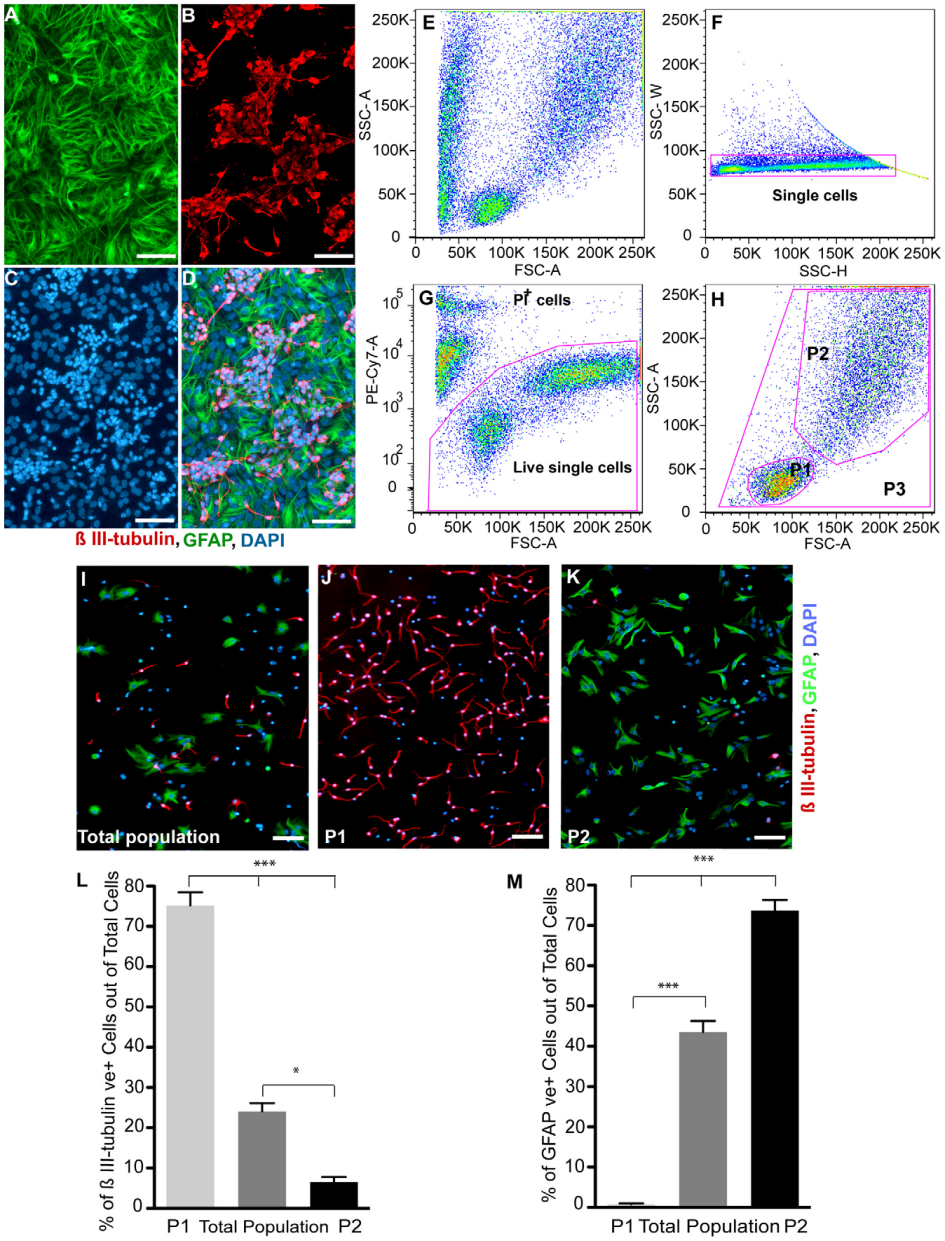
Differentiating neural stem cell progeny, cell sorting strategy and analysis of different populations post-sorting. (**A–D**): Representative micrographs of the neuroblast assay culture, 4 days after switching to growth factor free medium and stained for the astrocyte marker; GFAP (**A**), neuronal marker; β III-tubulin (**B**) and counterstained with DAPI(**C**). Notice the colonies of β III-tubulin positive neuronal cells on top of the astrocyte monolayer (**D**) and the nucleus size difference. (**E–H**): Cells were first plotted based on FSC, versus SSC (**E**) and then side scatter pulse width (SSC-W), versus side scatter pulse height (SSC-H) (**F**) to exclude doublets and clumps. After excluding dead or damaged cells based on Propidium Iodide (PI) uptake (**G**), cells were plotted based on FSC and SSC (**H**) and gates drawn to define 3 cell populations; P1 (FSC^low^ SSC^low^), P2 (FSC^high^ SSC^high^) and P3 (total cells). (**I–K**): Micrographs of sorted cells from P3 (**I**), P1 (**J**), and P2 (**K**) population that were stained (1 day after plating) for β III-tubulin, GFAP and DAPI. (**L–M**): The percentage of β III-tubulin IR (**L**) and GFAP IR (**M**) cells was quantified for each of the sorted populations. Comparing to the total cell population, the vast majority of the P1 population is composed of immature neurons; while very few are in the P2 population. Conversely, the P2 population is enriched with astrocytes, with few found in the P1 population. (mean±SEM; n = 4−9 independent experiments; * p<0. 05, *** p<0.0001, one-way ANOVA). Scale bars = 50 µm. Abbreviations: GFAP = Glial fibrillary acidic protein, IR = Immunoreactive, DAPI = 4′, 6-Diamidino-2-Phenylindole, FSC; Forward Scatter, SSC; Side Scatter, ANOVA; analysis of variances.

To isolate highly enriched immature neurons, we exploited the morphological differences observed between neurons and glia in differentiating NSC progeny. Using a modified protocol of Scheffler et al. (referred to as the neuroblast assay) [22], passaged NSCs were plated at high density in NSC medium containing 5% FCS, EGF and b-FGF. After 4 days, the growth factor containing medium was switched to a growth factor free medium and 4 days later the NBA exhibited colonies of small β III-tubulin IR cells on top of a flat GFAP IR cellular monolayer (Fig. 1A–D). Flow cytometry analysis of single cell suspensions from these differentiating NSC progeny revealed two separate populations (P1 & P2) based on their physical characteristics (Fig. S1) as defined by FSC and SSC plots (Fig. 1E and H). After exclusion of doublets or cell clumps using side scatter pulse width, versus side scatter pulse height (Fig. 1F) and dead cells as determined by propidium iodide (PI) uptake (Fig. 1G), analysis of the total live cells ([Fig. 1G] labeled as P3 in Fig. 1H) revealed that 24±2% of the cells were β III-tubulin IR (Fig. 1I and L), and 43.5±2.8% were GFAP IR (Fig. 1I and M). Using size and granularity, single viable cells were designed as being in the P1 (FSC^low^SSC^low^) or P2 (FSC^high^SSC^high^) population (Fig. 1H), sorted and plated in 96-well plates. One day later, dual staining for β III-tubulin and GFAP (Fig. 1 I–K,) demonstrated that 75.2±3.3% of the cells in the P1 population were β III-tubulin IR and 0.8±0.2% were GFAP IR (Fig. 1J, L, M). In contrast, in the P2 population (i.e. FSC^high^SSC^high^, Fig. 1H) 73.7±2.7% were GFAP IR and 6.6±1.2% were β III-tubulin IR (Fig. 1K, L, M). These results were confirmed by flow cytometry analysis using differentiating NSC cultures derived from doublecortin-GFP animal (Fig. S2A–O). In this case, virtually all (∼98%) of the GFP positive cells resided in P1 population (Fig. S2E-K) and 100% of sorted GFP positive cells expressed β III-tubulin (Fig. S2L-O). Hence, utilization of this strategy allows an enriched population of immature neurons (>70%) to be separated from astrocytes based on morphological characteristics using flow cytometry.

### Neurosphere and neural colony forming frequency of sorted cells

**Figure 2:**
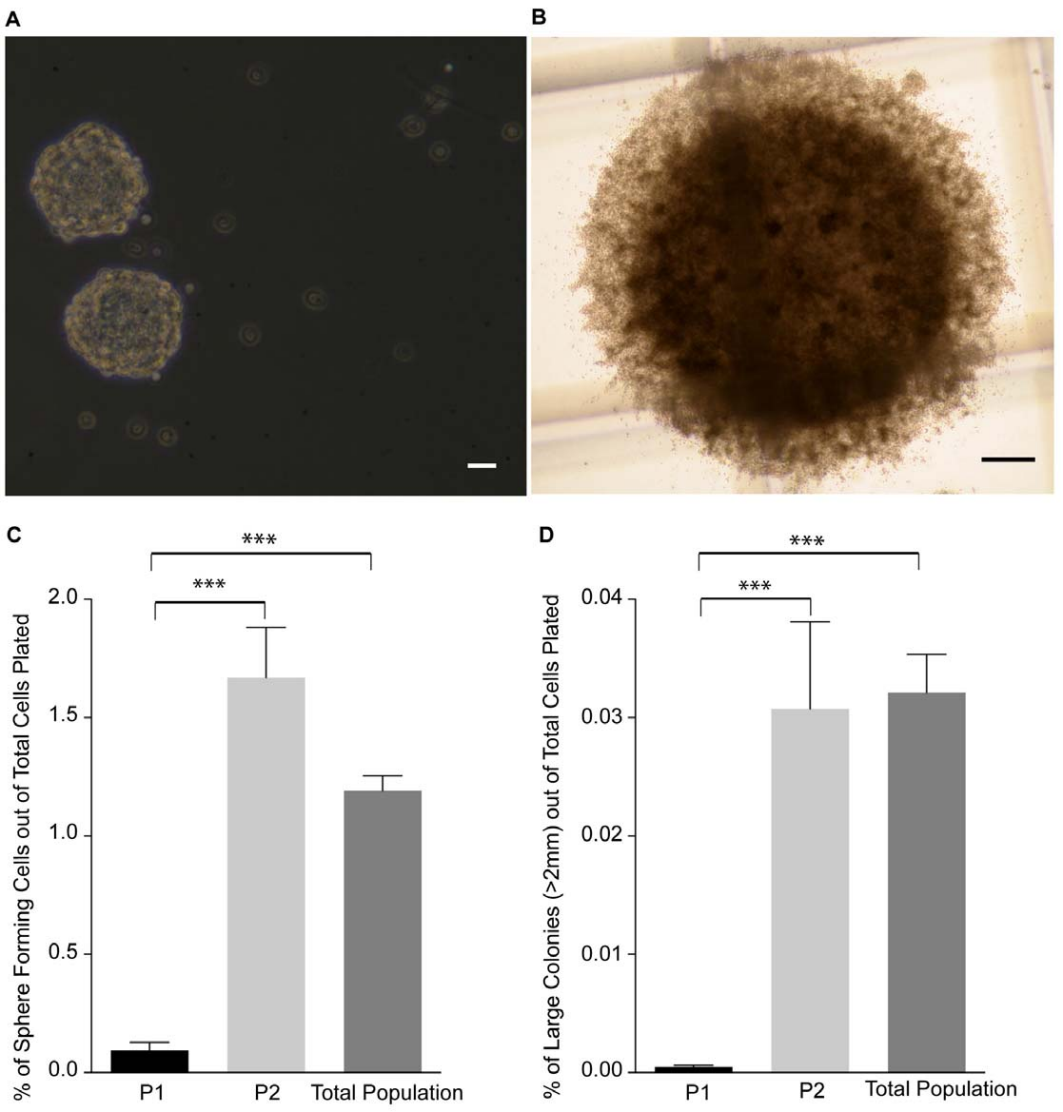
Frequency of stem and progenitor cells in the sorted populations using the NSA and N-CFCA. (**A**): Representative spheres from sorted cells, 7 days after plating in NSA culture. (**B**): Typical large colony (>2.0 mm) that is derived from a NSC, 21 days after plating in the N-CFCA. (**C–D**): Comparing the means of the sphere forming frequency in the NSA (**C**) and large colony (>2 mm) frequency in the N-CFCA (**D**) of cells sorted from the P1, P2 and P3 population. The majority of neural stem and progenitor cells can be removed from the neuronal cell population (P1), sorting based on FSC and SSC properties. (mean±SEM; n = 3−6 independent experiments; *** p<0.0001, one way ANOVA). Scale bars = 50 µm for **A** and 250 µm for **B**. Abbreviations: NSA = Neurosphere assay, N-CFCA = neural- colony forming cell assay.

NSC cultures are composed of a heterogeneous population of differentiated, progenitor and stem cells. While the potential for undesirable proliferation after implantation of NSCs is low [26], [17] the risk is still present [19], [20], [3]. Therefore to determine the precursor and stem cell frequency in our defined populations, we employed two assays: (1) Neurosphere Assay (NSA) that provides a readout of precursor cells (including both stem and progenitor cells) and (2) Neural-Colony Forming Cell Assay (N-CFCA) enabling quantitative discrimination between stem and progenitor cells [23]. Cells were isolated from three different populations (P1, P2 and P3) and cultured in the NSA and the N-CFCA for 7- and 21-days, respectively. In the NSA the numbers of spheres were counted (Fig. 2A and C) and the total number colonies, including large colonies (>2 mm in diameter), were enumerated in the N-CFCA (Fig. 2B and D). Within the P3 (the total) population sphere-forming frequency was 1.2±0.06% while the P1 and P2 population gave rise to 0.09±0.03 and 1.67±0.21%, respectively. Similarly, the colony-forming frequency was 1.5±0.2%, 0.07±0.02%, and 1.98±0.2% in the P3, P1 and P2 population, respectively. Hence, the vast majority (94.7±1.7%) of the neurosphere forming cells (i.e. stem & progenitor cells) resided in the P2 population (Fig. 2C). When plated in the N-CFCA the majority of cells with stem cell characteristics (colonies>2 mm) resided in the P2 population (0.031±0.007%, compared to the total population, 0.032±0.003%). Conversely, only a very small percentage of the P1 cells (0.0005±0.0001%) exhibited this defining stem cell feature (Fig. 2D). While, the NSC frequency in the total population was 1∶3114, it was almost exclusively contained within P2 population (1∶3250), with the NSC frequency of the P1 cells being quite rare (1∶200,000). Hence, using a sorting strategy based solely on FSC and SSC, nearly 95% of the proliferative precursors and greater than 96% of the bona fide NSCs could be eliminated from the NSC progeny thereby decreasing the probability of undesirable proliferation.

### Enrichment of immature neurons with negative selection

**Figure 3:**
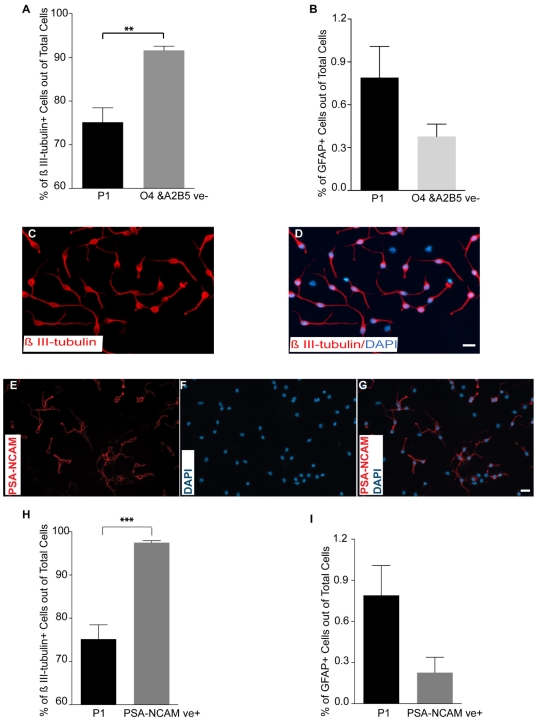
Negative and positive selection strategies to further improve neuronal cell enrichment. In order to achieve purer neuronal cell population, contaminating cells (O4 and/ A2B5+cells) were excluded from P1 population or PSA-NCAM^+^ cells were selected and isolated from the P1 population. (**A**): Comparison of the mean percentage of β III-tubulin IR cells from the sorted P1 vs. O4/A2B5 double excluded P1 cell population one day after plating. (mean±SEM; n = 5−9 independent experiments; ** p = 0.0038, Student's t-test,). (**B**): Comparison of the mean percentage of GFAP IR cells in sorted P1 vs. O4/A2B5 double excluded P1 cell population one day after plating. (mean±SEM; n = 5−9 independent experiments). (**C–D**): Micrographs from O4/A2B5 excluded P1 sorted cells showing that the majority of cells are β III-tubulin immunoreactive one day after sort. (**E–G**): PSA-NCAM staining on sorted cells from the P1 population one day after plating showing about 60% of sorted cells are PSA-NCAM IR. (**H–I**) Comparison of the mean percentage of β III-tubulin (**H**) and GFAP (**I**) positive cells in P1 and PSA-NCAM^+^ P1 sorted cells one day after plating (mean±SEM; n = 5−9 independent experiments, *** p = 0.0004, Student t-test,). Scale bars = 10 µm. Abbreviation: PSA-NCAM = Polysialic acid-neural cell adhesion molecule.

While encouraging, purification based on morphological characteristics alone achieved only 75% purity of the neuronal population. O_4_ and A_2_B_5_ (early oligodendroglial lineage markers [27]) immunocytochemistry was performed on the sorted P1 population and demonstrated contamination with immature glial cells (data not shown). Consequently, the P1 population was further enriched using a negative selection strategy. Double exclusion of O_4_ and A_2_B_5_ IR cells from the P1 population (Fig. S3A–F) increased the purity of the sorted β III-tubulin IR cells from 75.2±3.3% to 91.6±0.93% (Fig. 3A, C–D). The percentage of GFAP IR cells decreased from 0.8±0.2% in the sorted P1 population to 0.38±0.1% with negative selection, but did not reach statistical significance (Fig. 3B). The negative selection strategy with detection of specific surface markers for immature glial cells (O_4_ and A_2_B_5_) yielded a highly pure immature neuronal cell population.

### Isolation of immature neuronal cells based on PSA-NCAM immunoreactivity

While the negative selection resulted in a highly pure population (∼92%), we further evaluated a positive selection strategy using PSA-NCAM with the goal of obtaining increased purity. PSA-NCAM has been used to separate immature neuronal cells from E13.5 rat neural tubes [28] , to isolate neuronal precursor cells directly from adult mouse subventricular zone (SVZ) [29], and to purify ES cell derived neuronal cells using an immunopanning method [30]. Immunocytochemistry on sorted cells from the P1 population demonstrated PSA-NCAM immunoreactivity in approximately 60% of the cells (Fig. 3E–G) and appeared to be localized exclusively to immature neurons. Therefore, to obtain a more purified population of immature neurons, we isolated PSA-NCAM IR cells from the P1 population by flow cytometry (Fig. S3A–D and G, H). Using this approach, the purity of β III-tubulin IR cells reached near homogeneity, 97.5±0.44% (Fig. 3H). In addition, the mean percentage of GFAP IR cells in the PSA-NCAM^+^/P1 sorted cells decreased from 0.8±0.2% with sorting alone to 0.23±0.1% with positive selection, but did not reach statistical significance (Fig. 3I). In an attempt to reach higher purity, we sorted PSA-NCAM^+^ cells from O_4_ and A_2_B_5_ double excluded P1 cell population. The purity of β III-tubulin IR cells in this approach reached 97.7±0.27% but was not significantly different to the PSA-NCAM^+^/P1 sorted cells, 97.5±0.44% (Fig. S4). PSA-NCAM is expressed prior to β III-tubulin *in vivo*, which suggests that PSA-NCAM IR cells are more immature neuronal cells [28]. Therefore purification of PSA-NCAM cells from the P1 neuronal population allows for isolation of highly enriched and potentially a more developmentally synchronized immature neuronal subpopulation.

### Screening the survival effect of different factors on sorted immature neurons

**Figure 4:**
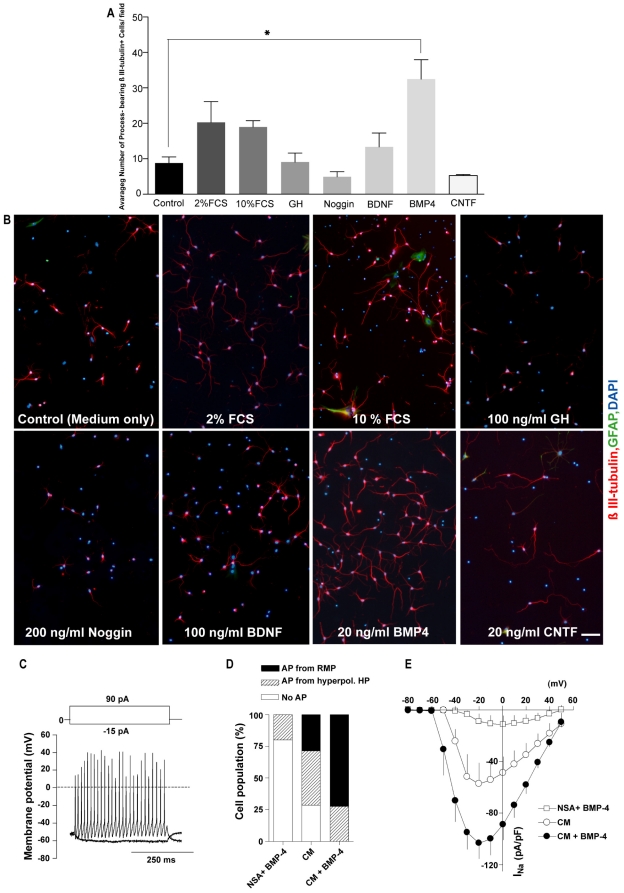
Sorted immature neurons survive *in vitro* and reach functional maturity. Immature neurons were sorted from P1 (FSC^low^ SSC^low^) population and plated in various culture conditions to find supportive factors that can increase their survival and functional maturity. (**A**): Comparison of the survival effects of different factors on the sorted immature neurons 6 days after plating (mean±SEM; n = 4 independent experiments, * P = 0.0205, one-way ANOVA on log transformed data). (**B**): Representative micrographs from different conditions on day 6 after plating. BMP4 treated condition showed the greatest number of process bearing β III-tubulin IR cells/field of view. (**C**): Depolarizing current injection-induced repetitive action potentials from a RMP of differentiating neurons cultured in astrocyte conditioned medium plus BMP-4 (20 ng/ml). (**D**): Effect of various culture conditions on action potential (AP) occurrence (n≥5). APs were evoked with depolarizing current injections, and cells were divided into three groups; cells with APs from a RMP, with APs only from a hyperpolarized holding potential (HP) of −60 mV and without APs. (**E**): I–V relationship of voltage-gated, tetrodotoxin-sensitive Na^+^ channel current of differentiating neurons under various culture conditions (n≥4). Series resistance (*Rs*) compensation was not applied. A voltage error of ∼10 mV was estimated for the maximum Na^+^ channel current amplitude given *Rs* of 2–5 MΩ. Scale bar = 50 µm. Abbreviations: RMP = resting membrane potential, BMP-4 = Bone morphogenetic protein-4.

*In vitro,* neuronal cells require support molecules or growth factors to enhance their survival and maturation [31]. Following sorting and plating in tissue culture dishes, the majority of the immature neurons died within a few days, likely due to the absence of appropriate support molecules. To address this dilemma, we tested seven compounds for their ability to promote survival. These factors included two concentrations of FCS (at 2% and 10%) and five growth factors (BDNF at 100 ng/ml, CNTF at 20 ng/ml, Noggin at 200 ng/ml, GH at 100 ng/ml and BMP-4 at 20 ng/ml (Fig. 4A, B). Growth factors were added once at the time of plating and cultures were fixed with 4% PFA and dual immunostained for β III-tubulin and GFAP after 6 days in culture. The numbers of DAPI-positive, process bearing β III-tubulin IR cells were evaluated as an indicator of surviving neurons. BMP4 treated immature neurons showed a statistically significant increase in the number of process bearing β III-tubulin IR cells/field of view (32.46±5.5) at 6 days after plating compared to the control (8.8±1.7) group (Fig. 4A, B). Serum, BDNF, CNTF, GH, and Noggin did not induce a significant change relative to controls. We confirmed the survival actions of BMP4 on our sorted neuronal cells by comparing the percentage of dead or dying cells labeled with PI (22.7±0.46% vs. 12.4±0.34%) and analyzed apoptotic cells as determined by total Annexin–V^+^/PI^+^ (24.5±0.3% vs. 12.1±0.2%). Furthermore, we investigated the percentage of cells undergoing apoptosis by comparing Annexin-V^+^/PI^-^ (4±0.56% vs. 2.2±0.23%) and anti-activated Caspase-3 immunoreactivity (3.7±0.3% vs. 2.3±0.1%) in control vs. BMP4 treated cultures, respectively (Fig. S5A–D). In all cases the addition of BMP4 (20 ng/ml) resulted in a significant reduction in cell death and apoptosis. It is possible that BMP4 can act as a mitogen for a small population of cells derived from the P1 population and increase the number of neurons by proliferation as opposed to survival, or in addition to survival. However, we feel this is unlikely for the following reasons: (1) BMP4 has not been reported to have mitogenic actions on immature neurons, (2) we observed a significant reduction in dead and dying cells (Fig. S5A–D) that supports the conclusion that BMP4 is having a survival effect, and (3) we have seen no overt signs of proliferation in these cultures in the presence of BMP4 (i.e. no clusters, doublets or signs of dividing cells). As we have not carried out any BrdU incorporation studies in the BMP4 treated cultures we cannot completely rule this out.

Taken together, these data demonstrate that sorted immature neuronal cell survival can be altered with the addition of appropriate growth factors and that the assay may be useful for identifying molecules that enhance the survival of immature neurons.

### Immunophenotyping of sorted immature neurons

The addition of BMP4 to the purified sorted neurons significantly enhanced survival and maturation, allowing us to investigate the neurotransmitter phenotype. Seven days after plating (and administration of BMP4 at 20 ng/ml on day 1 and 4) cells were fixed and dual immunostained for MAP-2 or NeuN (markers of mature neurons) and GAD65/67(GABAergic), DARPP-32 (GABAergic), tyrosine hydroxalase (TH-dopaminergic) or choline acetaly transferase (ChAT-cholinergic) to determine the neuronal phenotype. The majority of the cells (greater than 80%) expressed MAP-2 or NeuN suggesting the immature neurons were expressing a more mature phenotype (data not shown). The predominant neuronal phenotype for NSCs expanded as neurospheres is GABAergic [32], hence, it was not surprising that we saw no immunoreactivity for TH or ChAT but did observe that virtually all of the MAP-2 IR cells (i.e mature neurons) were GAD65/67 IR (98.35±1.5%). Similarly, all of the MAP-2 IR cells were DARPP-32 IR (99.8±0.1%), a marker of medium spiny neurons (Fig. S6A, B). Based on these results, neuronal cells sorted as described above, can survive and mature with the addition of BMP4, and are predominantly GABAergic medium spiny neurons.

While we noted a significant survival effect on the predominately GABAergic neurons in the BMP4 cultures, there was still a large amount of cell death. This suggests some degree of heterogeneity in the neuronal P1 population and that BMP4 does not have a survival effect on all GABAergic neurons derived from the Neurosphere Assay.

### Electrophysiological Properties of Sorted Neurons

Although immunocytochemistry suggested that the neurons would mature in culture, their utility for transplantation lay in their functional capabilities. Therefore, we investigated whether sorted immature neurons could reach functional maturation by using patch clamp electrophysiology. Three culture conditions were used: (1) complete NSC medium with BMP4, (2) astrocyte conditioned medium (CM-See the methods) and (3) CM plus BMP4. Following maturation under the three conditions for 2 weeks the cells were switched to a physiologic solution and electrical recordings obtained with patch electrodes connected to the cells and a patch amplifier. Electrophysiological findings showed that sorted immature neurons could undergo functional maturation *in vitro* if provided with supporting culture conditions (Fig. 4C–E). Table 1 summarizes the electrophysiological properties of differentiating neurons under the three maturation conditions tested. Although astrocyte conditioned medium facilitated functional maturation of sorted P1 immature neurons by increasing Na^+^ channel expression after two weeks in culture, it was not sufficient to evoke action potentials (APs) from the resting membrane potential (RMP). While seemingly mature based on marker expression, they did not function optimally. The addition of BMP4 (at 20 ng/ml on day 1,4 and 8) to the astrocyte conditioned medium induced further functional maturation as evidenced by APs at RMP, whereas BMP4 added to NSC medium alone had no significant effect on the occurrence of APs (Fig. 4D and E). Around 75% of the cells treated with a combination of conditioned medium and BMP4 exhibited repetitive APs from the RMP (Fig. 4D), which was probably due to increased Na^+^ and K^+^ channel current density and a slightly hyperpolarized RMP. Taken together, culturing the P1 sorted immature neurons in astrocyte-conditioned medium with BMP4 allows expression of mature neuronal markers and functional maturity. Furthermore, these cells could be used as a model to investigate the electrophysiological properties of maturing neurons.

**Table 1:** Electrophysiological properties of differentiating neurons.

	NSC medium+BMP-4	Conditioning medium (CM)	CM+BMP-4
Resting membrane potential (mV)	44.1±2.7 (5)	44.2±1.7 (7)	49.1±1.7 (11)
Input resistance (MΩ)	315±96 (5)	514±51 (7)	517±77 (11)
Membrane time constant (ms)	9.7±1.4 (5)	20.3±4.8 (7)	21.8±2.9 (11)
Membrane capacitance (pF)	37.0±5.9 (5)	38.1±6.0 (7)	46.5±6.3 (11)
Action potential amplitude (mV)	47.0 (1)	60.4±5.4 (5)	73.9±3.9 (11)
Action potential frequency (Hz)^a^	ND	37.6±1.3 (3)	35.3±4.7 (10)
Na^+^ channel current (pA/pF)^b^	−9.1±6.0 (4)	−57.4±16.2 (5)	−103.3±12.1 (8)† ***
K^+^ channel current (pA/pF)^c^	−133.4±43.6 (4)	−167.4±36.6 (5)	−238.0±52.5 (8)

Data are shown as mean±SEM, with number of cells analyzed in parentheses.

a Calculated from 1st and 2nd action potentials in response to a 90 pA depolarizing current step.

b Tetrodotoxin-sensitive transient inward current in response to a depolarizing voltage step of −20 mV from a holding potential (HP) of −80 mV.

c Tetraethylammonium-sensitive outward current in response to a depolarizing voltage step of +50 mV from a HP of −80 mV. ND; not determined.

***P<0.001; versus the NSC medium+BMP4 group.

† P<0.05; versus the CM group by one-way ANOVA.

### *In vivo* survival and maturation

**Figure 5:**
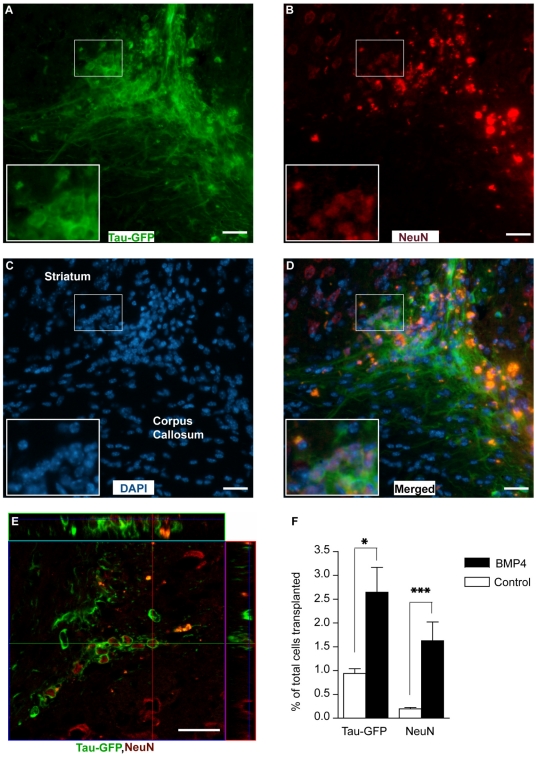
BMP-4 treatment of sorted P1 population improves their survival in adult mouse striatum. Freshly sorted cells P1 cells were resuspended in medium supplemented with 40 ng/ml of BMP-4 at a density of 5×10^4^ cells/µl (2 µl /animal) and injected into the right striatum of intact C57-BL6 mice and their survival was analysed 4 weeks later. (**A–D**): Representative micrographs from BMP4 group show that some of the implanted immature neurons survived and expressed the mature neuronal marker; NeuN. (**E**): Confocal micrograph from the same group shows some GFP-NeuN immunoreactive cells. (**F**): Bar graph shows the percentage of survived Tau-GFP and NeuN-IR cells in control and BMP4 groups as of total cells (1×10^5^ cells/animal) transplanted (mean±SEM; n = 4 animals/group, * P<0.05, ***P = 0.0002, Student's t-test). Scale bars = 20 µm. Abbreviation: NeuN = Neuronal nuclei.

We next assessed the ability of the sorted P1 neuronal cells to survive and mature following intracerebral implantation. P1 cells derived from a tau-GFP mouse were obtained as noted above, and implanted into the non-lesioned striatum of adult wild type C57/Bl6 mice. Four weeks after implanting 100 K cells, the brains of the transplanted animals were processed to evaluate the presence of GFP^+^/DAPI^+^ cells (i.e. viable transplanted cells). While significant amounts of autoflourescent dying or dead cells were identified, we found that a small percentage of donor cells survived, 0.94±0.1%, of which 20.1±2.6% were NeuN IR, or mature neurons (Fig. 5F and Fig. S7A–D). The majority of GFP IR cells were confined to the transplant core and fiber outgrowth was limited. In contrast, BMP4 treated P1 derived neurons showed an improved survival, 2.65±0.52% of total cells implanted, of which 60.2±4.2% percent were NeuN IR (Fig. 5A–F). In the BMP4 group, GFP IR cells exhibited more extensive outgrowth with processes being sent into the corpus callosum. As shown *in vitro*, upon maturation transplanted neuronal cells also acquired GABAergic phenotype by expressing GAD65/67 (Fig. S8A–C). We could not see any GFAP-IR cells amongst the surviving transplanted cells after 4 weeks implying that the immature neuronal cells had already chosen a neuronal fate and addition of BMP-4 together with implantation did not alter their fate (Fig. S9A–C). Hence, enriched NSC-derived neuronal population are capable of surviving and differentiating into mature neuronal cells after transplantation if provided with appropriate supporting factors such as BMP4.

### *In vivo* proliferation capability of P1 population

**Figure 6:**
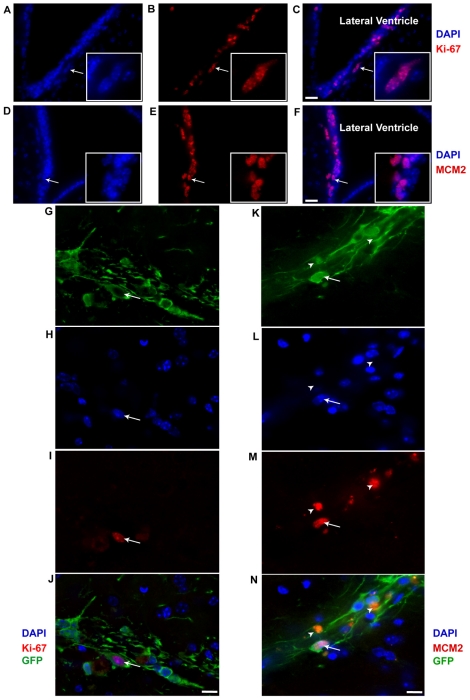
Representative confocal micrographs of sorted Tau-GFP positive immature neurons (P1 population) in NOD-SCID mouse striatum. (**A–C**): Ki-67 and (**D–F**): MCM_2_ immunoreactive cells of the subventricular zone serve as an internal positive control. Co-localization of immunoreactive cells with DAPI shows that Ki67 and MCM2 are exclusively nuclear staining. (**G–J**) Ki-67 staining in transplant region shows a Ki-67^+^/GFP^−^ cell (*arrow*). (**K–N**) MCM2 staining in transplant region shows a MCM_2_^+^/GFP^+^ cell (*arrow*) and two GFP^+^ cells that do not have DAPI^+^ nuclei but show red fluorescence (*arrow head*). The auto-fluorescent figures are probably clumps of dying or dead cell that show auto-fluorescence even before staining. Scale bars = 20 µm for **A–F**, and 10 µm for **G–N**. Abbreviations: GFP = Green fluorescent protein, MCM2 = Mini-chromosome maintenance protein.

Although the cells were found to survive *in vivo*, the potential for cell proliferation had only been tested *in vitro* with the NSA and N-CFCA under conditions that drive growth of precursor cells (i.e. growth factor supplemented medium). To evaluate *in vivo* proliferative potential of sorted immature neurons from P1 population, we transplanted freshly sorted P1 cells from a tau-GFP mouse into the striatum of immunodeficient mice. Four weeks after implantation into NOD-SCID animals, in contrast to the active proliferative zones like the subventricular area (Fig. 6A–F), only a small fraction of the surviving GFP IR cells were positive for Ki-67 and MCM2. While 0.21±0.12% GFP^+^/DAPI^+^ cells were Ki67 immunoreactive (Fig. 6G–J), 0.29±0.17% were positive for MCM2 (Fig. 6K–N). Hence, the sorted P1 population exhibited little *in vivo* proliferation activity.

## Discussion

This work is an attempt to purify immature neuronal cells from a renewable source of NSC progeny based on their size and internal complexity. Our findings show that immature neurons have very small cell bodies and nuclei, and are distinguishable from astrocytes using microscopy (Fig. 1A–D and Fig. S1A–C). Exploiting these characteristics via flow cytometry, where distinct cell populations can be separated based on light scatter properties, we obtained an enriched immature neuronal population that developed a GABAergic phenotype and was minimally contaminated with glial cells. Further enrichment, to near homogeneity (97%), was achieved based on a negative (eliminate O4+ & A2B5+ cells) or positive (PSA-NCAM^+^) selection. These immature neurons are sensitive to their environmental conditions and are not very robust, however, their survival and maturation, both *in vitro* and *in vivo*, could be enhanced by exposure to BMP4.

Neuronal cells have been enriched from primary brain tissue based on cell size [33], transgene [31] and PSA-NCAM expression [29]. While these approaches provide enriched populations of cells for pre-clinical studies, they are limited in their clinical application due to supply and ethical issues of harvesting donor tissue from primary human cells. ES cells represent a viable alternative, and flow cytometry has been used together with transgene expression to derive purified neuronal populations with attenuated tumor forming ability [34], [35]. This has also been combined with immunopanning for PSA-NCAM to further enrich the neuronal population [30]. Although potentially useful as a renewable source of cells, genetic alteration of donor cells is less than ideal and ES cells carry an inherent risk of tumor formation that may be unacceptable for wide clinical application. More recently, induced Pluripotent Stem Cells (iPS) [36] have been used to generate neural cells [37] that may serve as a more ethically acceptable autologous donor population, in particular when induced through non-genetic methods via a transient neural stem cell population [38].

One of the risks in using any renewable precursor population as a donor source of cells is the potential for uncontrolled or unwanted proliferation. Recently, it has been proposed that NSC progeny are the *in situ* source of gliomas [39] and that extensive culture of adult stem cells may generate malignant phenotypes [40], [41] While this is countered by a detailed study of extensively passed adult NSCs demonstrating their genetic stability, resilience to transformation and inability to form tumors in immunocompromised mice [17], a report by Amariglio and colleagues of a pediatric patient who had received repeated intracerebellar and intrathecal injections of large numbers of primary fetal CNS cells, and presented four years later with a well differentiated benign glioneuronal neoplasm, suggests a more cautious approach. While the tumor was likely the result of the large number of neural progenitor cells [42], the presence of NSCs within the slurry of donor cells and the resultant undesired growth (although not transformed nor malignant) raises concern and the need for more defined populations of cells for transplantation. Our sorted P1 population was not only highly enriched with immature neuronal cells but also contained a precursor population (include stem cells and progenitor cells) of approximately 1∶1111 cells, which contrasts the 1∶83 precursor cell frequency in the overall population, representing a 13 fold reduction. More importantly though was the change in the bona fide stem cell population (based on the N-CFCA [23]) where its frequency was reduced from 1∶3114 to 1∶200,000, a 64-fold change. While we were unable to eliminate all of the stem or precursor cells, the reduction may be functionally important. To test this we implanted 100,000 cells from our P1 population (derived from a tau-GFP transgenic) into NOD/SCID animals and 4-weeks later counted the number of DAPI+/GFP expressing cells that were immunoreactive for Ki-67 or MCM2. A very small population of cells was found to express Ki-67 or MCM2 (0.21±0.12% and 0.29±0.17%, respectively), revealing that our sorting procedure did not eliminate all cells witha proliferative potential. This contrasted the results of Yan et al.[19] who reported that 3–5% of their NSC progeny expressed Ki-67, 3–6 months post-implantation. While a small fraction of cells retained the ability to proliferate we observed no clusters of proliferation, proliferative rosettes or masses of cells that would indicate delirious uncontrolled growth, rather the few cells we noted (1 out of 400) were randomly scattered around the remaining surviving cells. It is important to note that these data were generated using only the P1 population (FSC^low^SSC^low^) without additional negative or positive selection and a further reduction in the number of dividing cells may be achieved by employing these additional purification strategies.

Our findings demonstrated that addition of BMP4 (at 20 ng/ml) to our sorted immature neurons increases their survival and is consistent with the report by Lim and colleagues [43] who reported that BMP4 increased the survival of purified immature neuroblasts from dissociated SVZ cultures. Furthermore, it has been shown that in the olfactory bulb, high concentration of BMP4 inhibits proliferation of neuronal progenitors, promotes their exit from the cell cycle and initiates differentiation. In addition, BMP4 promotes glial differentiation of progenitor cells from the forebrain subventricular zone [44] and CNS derived tumor cells [45].

Treatment of sorted P1 population with BMP4 did not increase the number of GFAP IR cells after six days of culture (data not shown), nor did we observe any GFAP positive cells 4 weeks after transplantation into the adult striatum of BMP4 treated P1 cells. This suggests the P1 cells are committed to neuronal fate and that BMP4 enhances their maturation and survival as demonstrated by an increase in the percentage of NeuN IR cells in BMP4 treated transplanted P1 cells, relative to controls. Our electrophysiological data support the role of BMP-4 in supporting functional maturation (showing action potential from RMP or establishment of Na^+^ channels in cell membrane).

Application of this methodology for purification of a defined population of highly enriched immature neuronal cells from a heterogeneous population of human NSC progeny provides a renewable source of cells that may have potential for *in vitro* studies such as drug screening, neurotoxicolgy, electrophysiology and to act as a source of cells for implantation to repair the injured CNS. However, additional work still needs to be carried out to determine if the purified implanted donor cells are able to functionally integrate. There are many research studies showing therapeutic application of neural stem and progenitor cells in animal models of diseases including reduction in seizure activity [46], [47], alleviating neuropathic pain and restoring disrupted neuronal circuitry in spinal cord injury [48], [49], increasing axonal sprouting in neonatal hypoxic ischemic brain injury [50], and delaying onset and progression of amyotrophic lateral sclerosis (ALS) [51]. Applying the methodology developed in this study to human neural stem cells, we hope to be able to define the starting population of donor cells (e.g. 60% neurons, 40% glia or 90% neurons and 10% glia) which may provide the opportunity to determine the cell type(s) responsible for a phenotypic change, to investigate the potential mechanisms, and adjust dosing strategies based on a particular disease or disease stage. Application of supportive factors that increase survival of the immature neurons after transplantation, in particularly when used together with a permissive vehicle such as a 3-dimensional matrix, may increase the practical application of NSC as a source of donor material for cell replacement therapies.

## Supporting Information

Figure S1 **Representative micrographs show sorted cells with different morphologies.** (**A–C**): Sorted neurons (P1 population) (**A)** have a small cell body and nucleus, relative to astrocytes (P2 population) (**B**) which have a larger cell body and nucleus. Oligodendrocytes (**C**) have a wider range of sizes in their cell body and nucleus comparable to those of astrocytes and neuronal cells and are scattered both in P1 and P2 population. Scale bars = 10 µm.
(TIF)

Figure S2 **Representative micrographs and sort plots for differentiating doublecortin-GFP neural stem cell progeny.** (**A–D**): Micrographs of neuroblast assay on day 4 after switching to growth factor free medium; Phase (**A**), Doublecortin-GFP (**B**), β III-tubulin immunostained (**C**) and Merged micrograph (**D**). Notice the colonies of β III-tubulin positive neuronal cells (**A–D**) and co-localization of β III-tubulin and GFP- IR cells (**D**). (**E–K**): Cells were first plotted based on FSC, versus SSC (**E**) and then side scatter pulse width (SSC-W), versus side scatter pulse height (SSC-H) (**F**) to exclude doublets and clumps. After excluding dead or damaged cells based on PI uptake (**G**), cells were plotted using FSC and SSC (**H**) to gate the P1 (FSC^low^ SSC^low^), P2 (FSC^high^ SSC^high^) and P3 (total) populations. Control gates were set using non-GFP expressing, wild-type control cultures in the NeuroBlast Assay (**I**) and then applied to doublecortin-GFP cells to locate positive staining cells (**J**). Back-gating doublecortin-GFP cells in a FSC and SSC plot revealed that almost all (98%) of GFP+ cells are in P1 population suggesting that immature neuronal cells can be isolated from the overall population based on FSC and SSC properties. (**L–O**): Representative micrographs of sorted GFP cells demonstrating that 100% of GFP IR cells (**l**) were β III-tubulin IR confirming their neuronal identity (**M–O**). Scale bars = 20 µm. Abbreviation: PI = Propidium Iodide.
(TIF)

Figure S3 **Negative and positive flow cytometry selection approaches to increase neuronal cell enrichment.** In order to increase neuronal purity, contaminating cells (O4 and/ A2B5+cells) were excluded from P1 population or PSA-NCAM^+^ cells were selected and sorted from the P1 population. (**A–D**): Cells were first plotted based on FSC, versus SSC (**A**), then side scatter pulse width (SSC-W), versus side scatter pulse height (SSC-H) to exclude doublets and clumps (**B**). After excluding dead or damaged cells based on Propidium Iodide (PI) uptake **(C**), single viable cells were plotted based on FSC and SSC **(D**) and gates set to define the P1 (FSC^low^ SSC^low^), and P2 (FSC^high^ SSC^high^) populations. From the P1 population, appropriate gates were set using a negative control sample (Isotype control antibody) (**E, G**). O4 and A2B5, were used to identify glial precursors in the P1 population (**F**), while PSA-NCAM was used to identify the immature neurons (**H**) within the P1 population.
(TIF)

Figure S4 **Sorting PSA-NCAM+ cells from P1 population versus PSA-NCAM+ cells from O4&A2B5 double excluded P1 population.** Comparison of the mean percentage of β III-tubulin IR cells in sorted PSA-NCAM^+^ cells from P1 vs. sorted PSA-NCAM^+^ cells from O4/A2B5 double excluded P1 cell population one day after plating. This data shows that combining negative (O4&A2B5 double exclusion) and positive (PSA-NCAM^+^ cell selection) sorting strategies does not further increase the purity of neuronal cells comparing to sorting PSA-NCAM^+^ cells directly from P1 cell population (mean±SEM; n = 3−5 independent experiments).
(TIF)

Figure S5 **Cell death in control versus BMP4 treated immature neuronal cells.** (**A–D**): One day after plating in medium only or medium supplemented with 20 ng/ml of BMP4, sorted P1 cells were labeled for Annexin-V, active caspase-3, β-III tubulin and PI and the percentage of dead or dying cells was quantified using flow cytometry. (**A**) Percentage of PI^+^ cells is significantly reduced in the BMP4 treated cultures (mean±SEM; n = 4−6 experiments/condition; ***p<0.0001). (**B**) Similarly the percentage of cells staining positive for Annexin-V is significantly reduced in the BMP4 treated cells (mean±SEM; n = 4**−**6 experiments/condition; ***p<0.0001,). (**C**) A comparison of live cells (PI^-^) find a significant reduction in Annexin-V binding in the BMP4 containing cultures (mean±SEM; n = 4−6 experiments/condition; **p<0. 05), (**D**) while an analysis of the β-III-tubulin expressing cells finds that the addition of BMP4 produces a significant reduction in the percentage of cells immunoreactive for activated caspase-3 (an early marker of apoptosis) (mean±SEM; n = 5 experiments/condition; ** p<0. 05). Together, these data support the conclusion that BMP4 has a survival effect on immature neurons sorted from the P1 population. Abbreviations: PI = Propidium Iodide.
(TIF)

Figure S6 **Immunophenotyping of sorted immature neurons.** P1 sorted neuronal cells were differentiated in astrocyte conditioned medium supplemented with 20 ng/ml of BMP-4 in order to identify their phenotype. (**A**): Representative micrographs from differentiated P1 sorted neuronal cells 7 days after plating. (**B**): Graph showing the percentage of GAD 65/67 and DARPP-32 IR cells out of total MAP-2 IR cells after 7 days differentiation in astrocyte conditioned medium supplemented with 20 ng/ml BMP4 (mean±SEM, n = 3 independent experiments). No other neuronal phenotypes (i.e. Dopaminergic, Cholinergic) were detected. Scale bars = 10 µm. Abbreviations: BMP-4 = Bone morphogentic protein-4, GAD 65/67 = Glutamic acid decarboxylase 65/67, DARPP-32 = Dopamin and cAMP regulated phosphoprotein-32, Map-2 = Microtubule associated protein-2.
(TIF)

Figure S7 **Sorted P1 cells implanted into the intact adult mouse striatum.** Freshly sorted cells from P1 population were resuspended in NSC medium at a density of 5×10^4^ cells/µl and injected (2 µl /animal) into the right intact striatum of C57-BL6 mice. Cell survival and maturation was analysed 4 weeks later. (**A–D**): Representative micrographs from the transplant region demonstrated that the majority of the implanted cells (GFP+) (**A**) And approximately 1% of the implanted cells (*arrows*) survived, of which roughly 20% expressed NeuN (**B–D**), a mature neuronal marker (see figure 5F for quantitative data). Scale bar = 20 µm.
(TIF)

Figure S8 **Sorted P1 cells give rise to GABAergic neurons after implantation into the adult mouse striatum.** (**A–C**) Representative confocal micrographs illustrate that implanted P1 neuronal cells (**A**, green) that were treated with BMP4 survive and acquire GABAergic phenotype by expressing GAD65/67 (**B**; red and **c**, merged) 4 weeks post implantation. Scale bar = 20 µm.
(TIF)

Figure S9 **Sorted P1 cells do not differentiate into astrocytes after implantation into the adult mouse striatum.** (**A–C**): Representative confocal micrographs show that implanted BMP4 treated P1 neuronal cells (**A**, green) survive but do not differentiate into GFAP expressing cells (**B**, red) as evident in the merged picture (**C**). Scale bar = 20 µm.
(TIF)
