# Supplementary figures and images for: Purification of Immature Neuronal Cells from Neural Stem Cell Progeny
Source: PLoS One. 2011 Jun 3;6(6):e20941. doi: 10.1371/journal.pone.0020941 (PMC3109004; doi:10.1371/journal.pone.0020941)

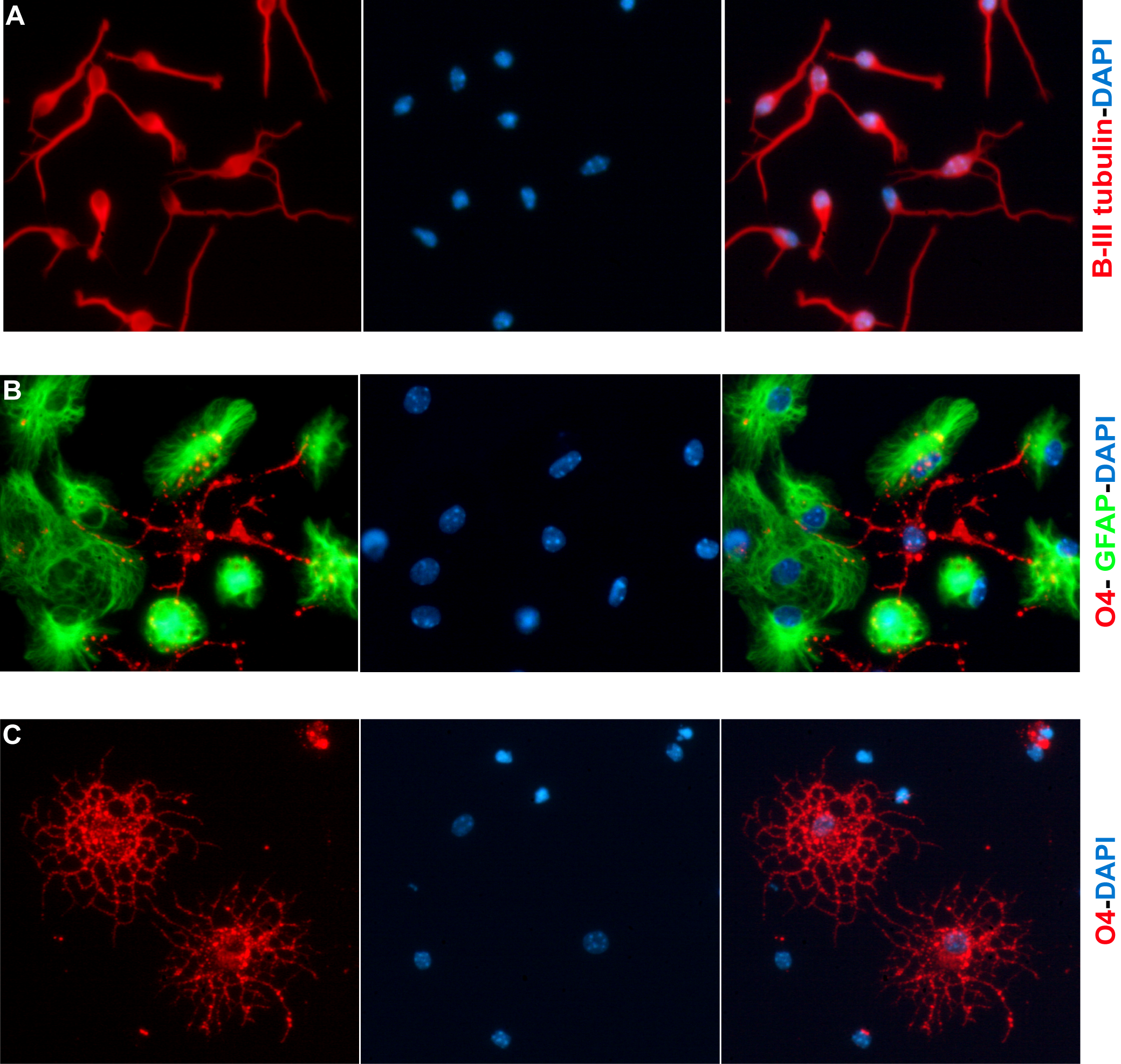

Supplement: Figure S1 — Representative micrographs show sorted cells with different morphologies. (A–C): Sorted neurons (P1 population) (A) have a small cell body and nucleus, relative to astrocytes (P2 population) (B) which have a larger cell body and nucleus. Oligodendrocytes (C) have a wider range of sizes in their cell body and nucleus comparable to those of astrocytes and neuronal cells and are scattered both in P1 and P2 population. Scale bars = 10 µm. (TIF) [file pone.0020941.s001.tif]

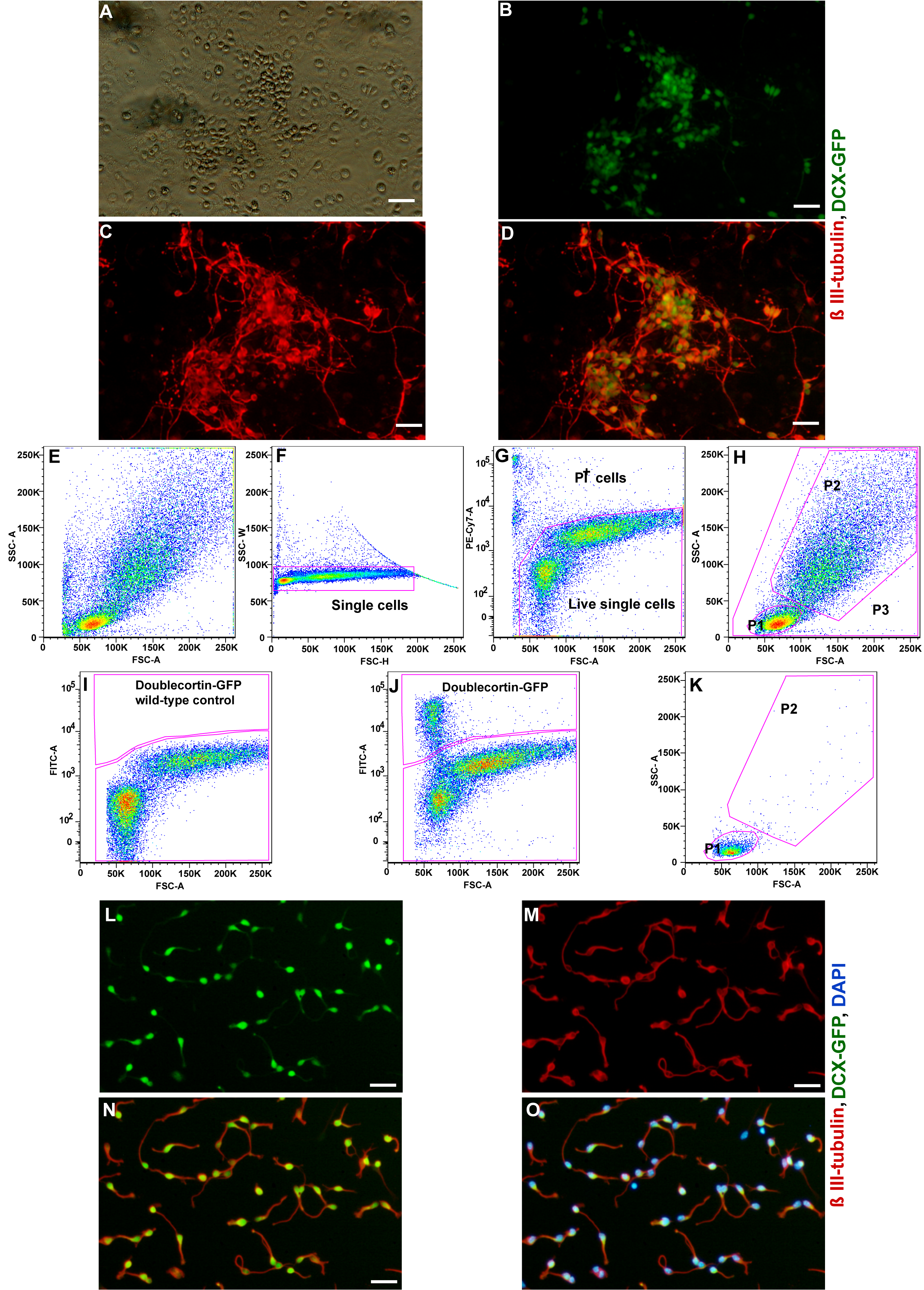

Supplement: Figure S2 — Representative micrographs and sort plots for differentiating doublecortin-GFP neural stem cell progeny. (A–D): Micrographs of neuroblast assay on day 4 after switching to growth factor free medium; Phase (A), Doublecortin-GFP (B), β III-tubulin immunostained (C) and Merged micrograph (D). Notice the colonies of β III-tubulin positive neuronal cells (A–D) and co-localization of β III-tubulin and GFP- IR cells (D). (E–K): Cells were first plotted based on FSC, versus SSC (E) and then side scatter pulse width (SSC-W), versus side scatter pulse height (SSC-H) (F) to exclude doublets and clumps. After excluding dead or damaged cells based on PI uptake (G), cells were plotted using FSC and SSC (H) to gate the P1 (FSClow SSClow), P2 (FSChigh SSChigh) and P3 (total) populations. Control gates were set using non-GFP expressing, wild-type control cultures in the NeuroBlast Assay (I) and then applied to doublecortin-GFP cells to locate positive staining cells (J). Back-gating doublecortin-GFP cells in a FSC and SSC plot revealed that almost all (98%) of GFP+ cells are in P1 population suggesting that immature neuronal cells can be isolated from the overall population based on FSC and SSC properties. (L–O): Representative micrographs of sorted GFP cells demonstrating that 100% of GFP IR cells (l) were β III-tubulin IR confirming their neuronal identity (M–O). Scale bars = 20 µm. Abbreviation: PI = Propidium Iodide. (TIF) [file pone.0020941.s002.tif]

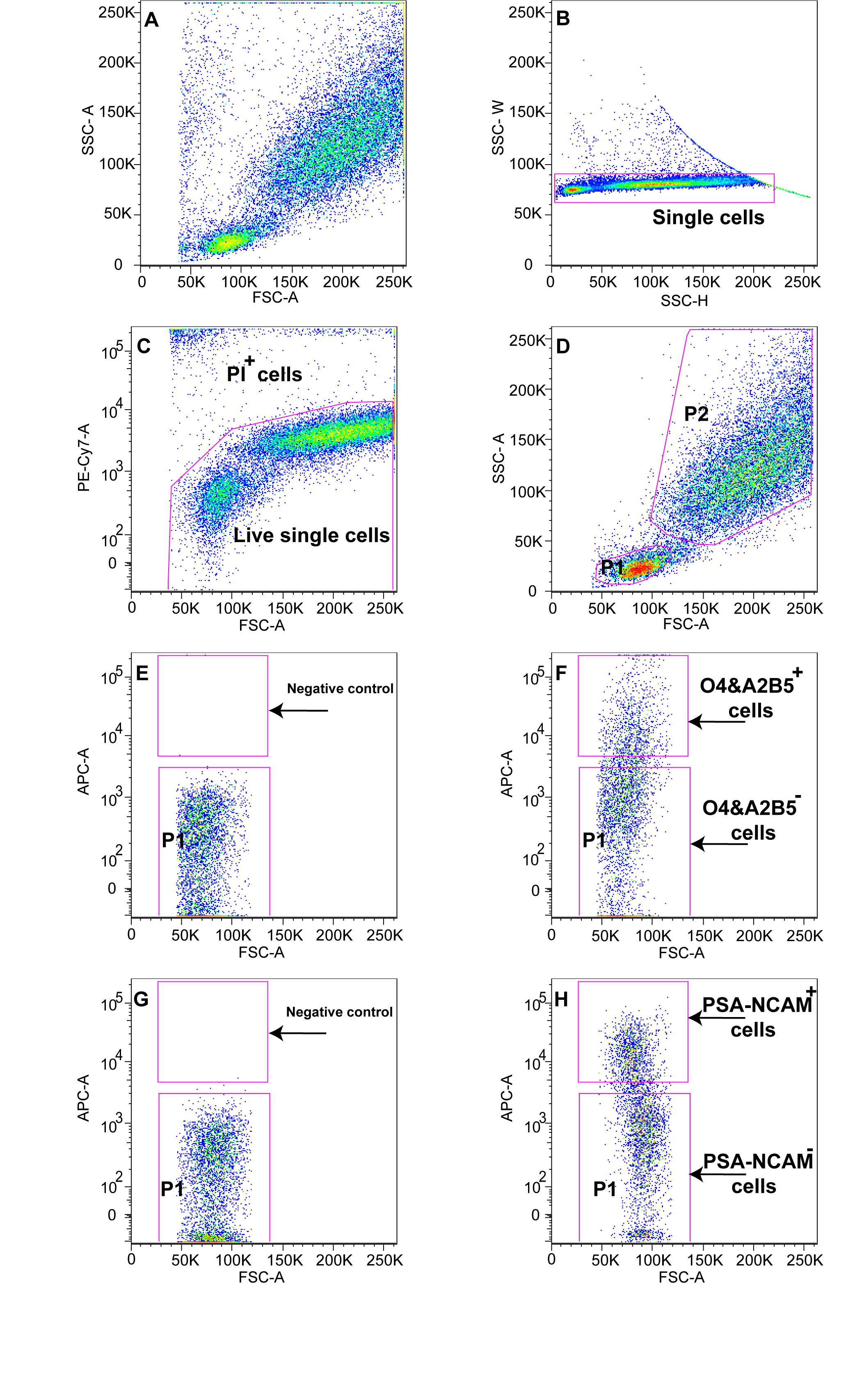

Supplement: Figure S3 — Negative and positive flow cytometry selection approaches to increase neuronal cell enrichment. In order to increase neuronal purity, contaminating cells (O4 and/ A2B5+cells) were excluded from P1 population or PSA-NCAM+ cells were selected and sorted from the P1 population. (A–D): Cells were first plotted based on FSC, versus SSC (A), then side scatter pulse width (SSC-W), versus side scatter pulse height (SSC-H) to exclude doublets and clumps (B). After excluding dead or damaged cells based on Propidium Iodide (PI) uptake (C), single viable cells were plotted based on FSC and SSC (D) and gates set to define the P1 (FSClow SSClow), and P2 (FSChigh SSChigh) populations. From the P1 population, appropriate gates were set using a negative control sample (Isotype control antibody) (E, G). O4 and A2B5, were used to identify glial precursors in the P1 population (F), while PSA-NCAM was used to identify the immature neurons (H) within the P1 population. (TIF) [file pone.0020941.s003.tif]

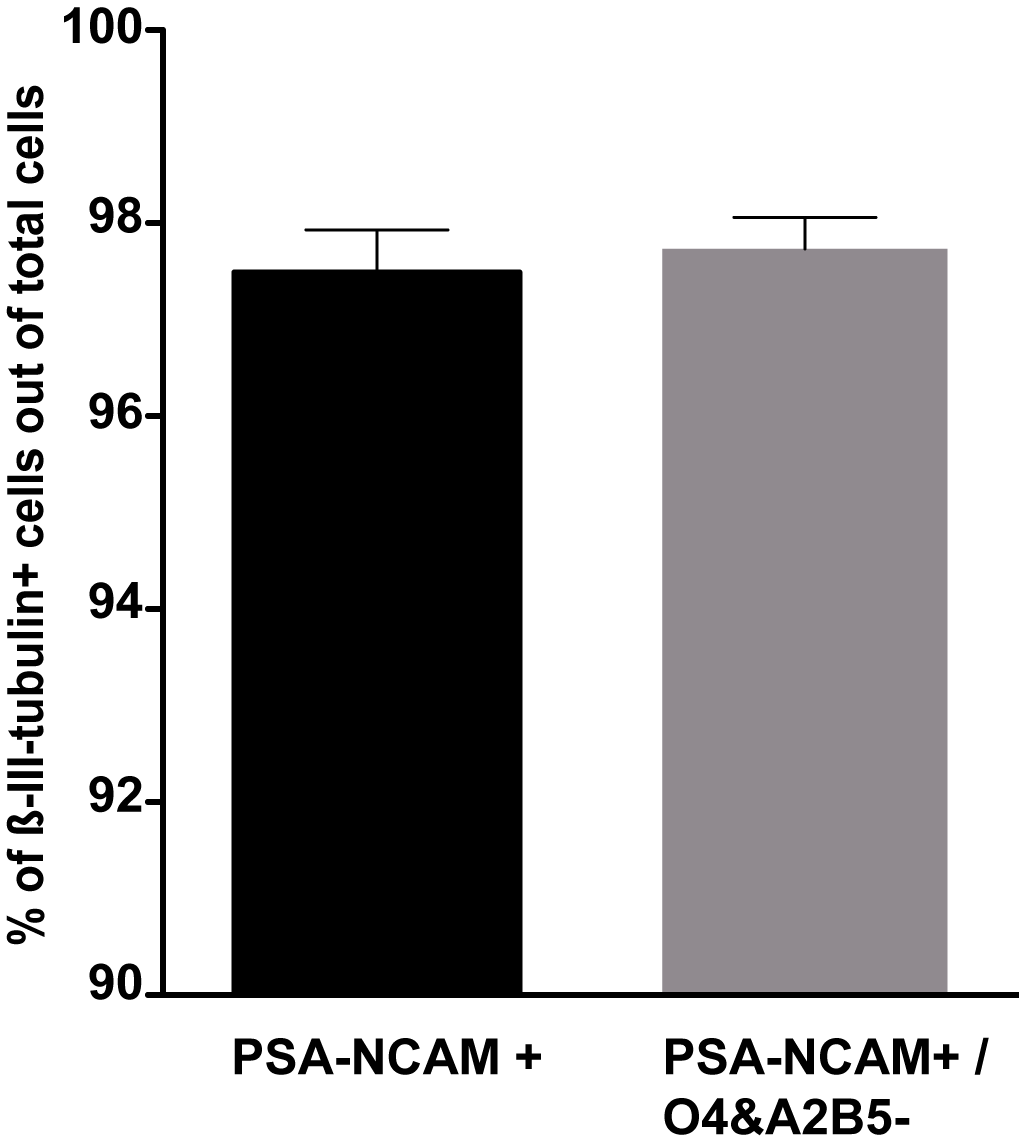

Supplement: Figure S4 — Sorting PSA-NCAM+ cells from P1 population versus PSA-NCAM+ cells from O4&A2B5 double excluded P1 population. Comparison of the mean percentage of β III-tubulin IR cells in sorted PSA-NCAM+ cells from P1 vs. sorted PSA-NCAM+ cells from O4/A2B5 double excluded P1 cell population one day after plating. This data shows that combining negative (O4&A2B5 double exclusion) and positive (PSA-NCAM+ cell selection) sorting strategies does not further increase the purity of neuronal cells comparing to sorting PSA-NCAM+ cells directly from P1 cell population (mean±SEM; n = 3−5 independent experiments). (TIF) [file pone.0020941.s004.tif]

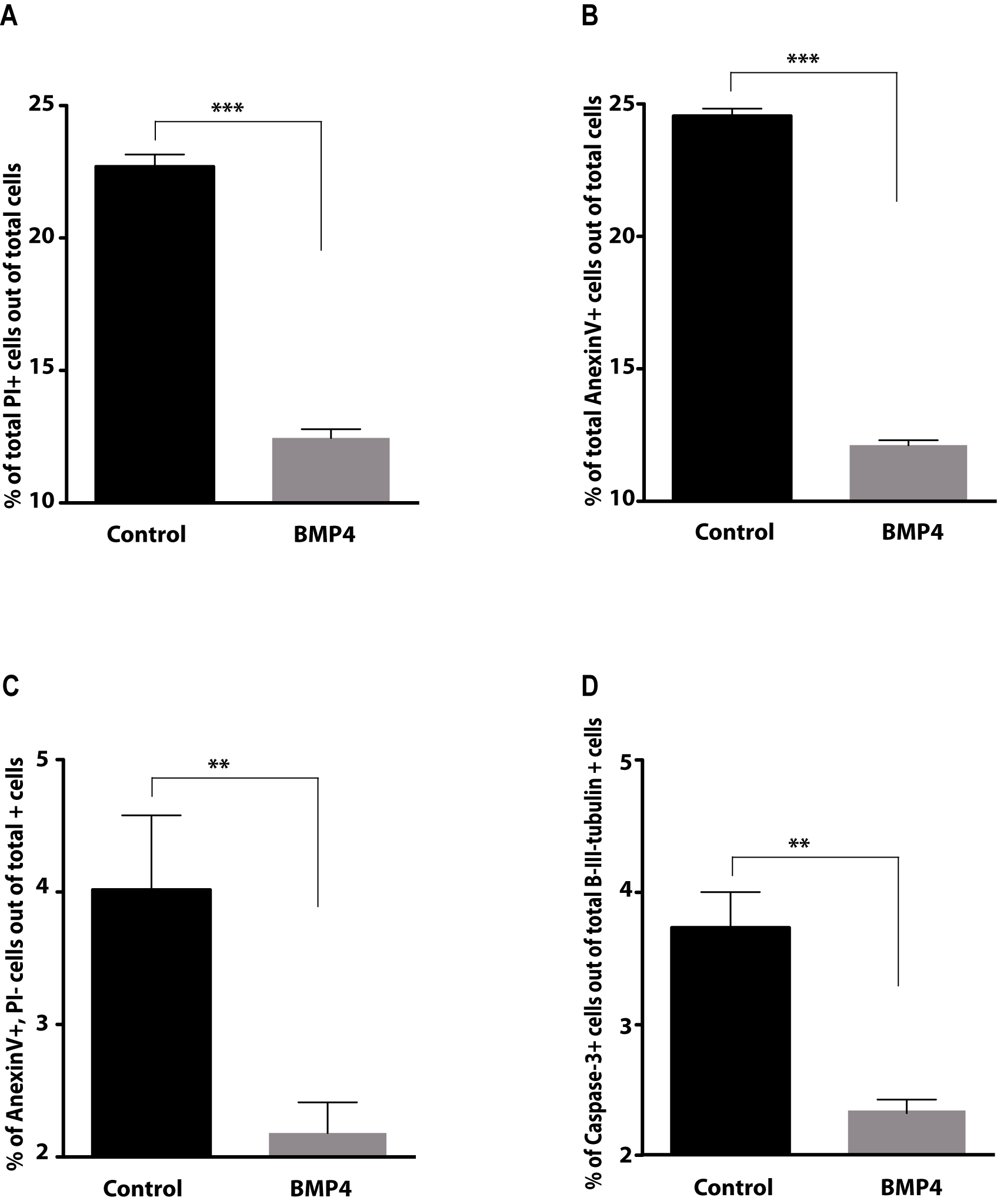

Supplement: Figure S5 — Cell death in control versus BMP4 treated immature neuronal cells. (A–D): One day after plating in medium only or medium supplemented with 20 ng/ml of BMP4, sorted P1 cells were labeled for Annexin-V, active caspase-3, β-III tubulin and PI and the percentage of dead or dying cells was quantified using flow cytometry. (A) Percentage of PI+ cells is significantly reduced in the BMP4 treated cultures (mean±SEM; n = 4−6 experiments/condition; ***p<0.0001). (B) Similarly the percentage of cells staining positive for Annexin-V is significantly reduced in the BMP4 treated cells (mean±SEM; n = 4−6 experiments/condition; ***p<0.0001,). (C) A comparison of live cells (PI-) find a significant reduction in Annexin-V binding in the BMP4 containing cultures (mean±SEM; n = 4−6 experiments/condition; **p<0. 05), (D) while an analysis of the β-III-tubulin expressing cells finds that the addition of BMP4 produces a significant reduction in the percentage of cells immunoreactive for activated caspase-3 (an early marker of apoptosis) (mean±SEM; n = 5 experiments/condition; ** p<0. 05). Together, these data support the conclusion that BMP4 has a survival effect on immature neurons sorted from the P1 population. Abbreviations: PI = Propidium Iodide. (TIF) [file pone.0020941.s005.tif]

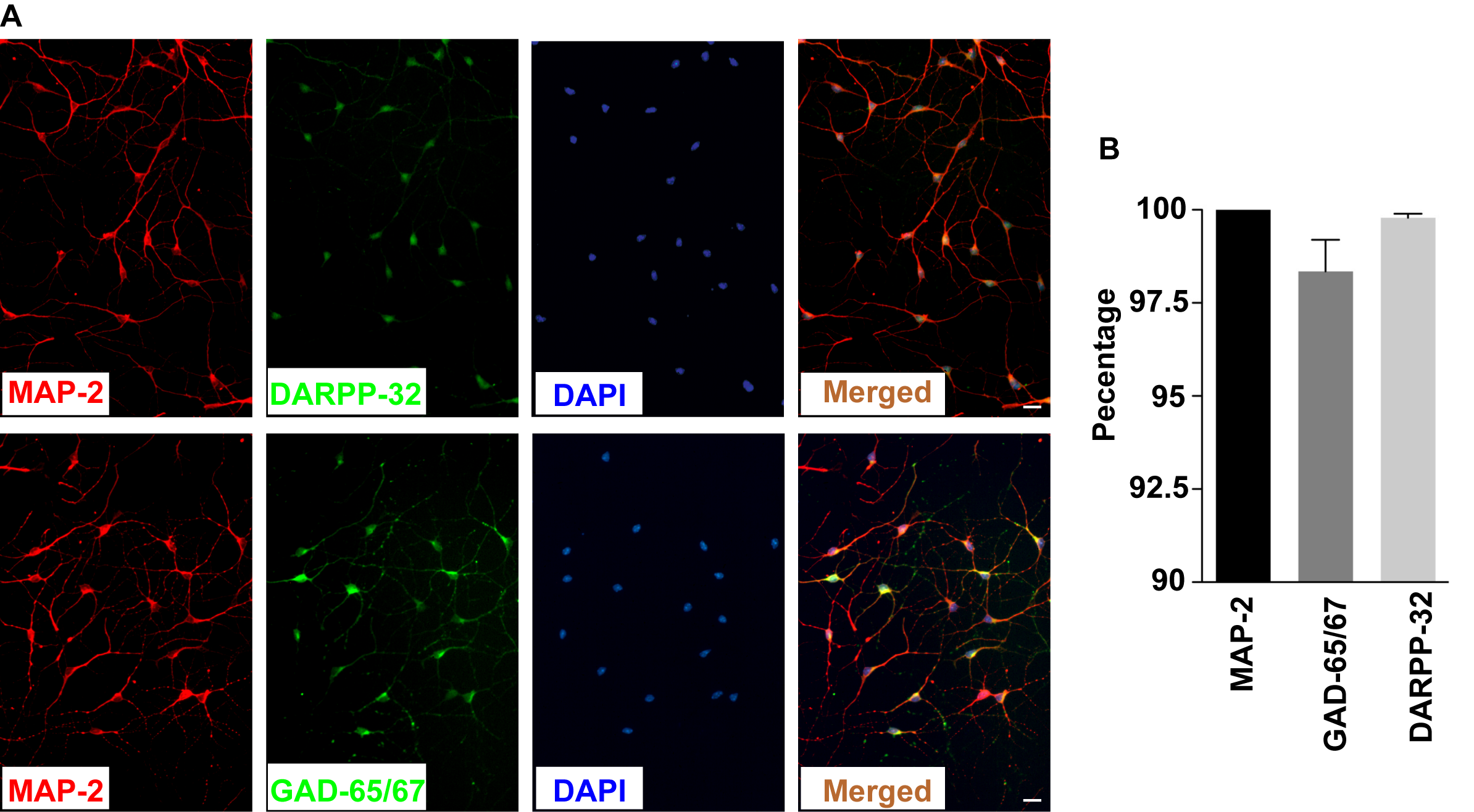

Supplement: Figure S6 — Immunophenotyping of sorted immature neurons. P1 sorted neuronal cells were differentiated in astrocyte conditioned medium supplemented with 20 ng/ml of BMP-4 in order to identify their phenotype. (A): Representative micrographs from differentiated P1 sorted neuronal cells 7 days after plating. (B): Graph showing the percentage of GAD 65/67 and DARPP-32 IR cells out of total MAP-2 IR cells after 7 days differentiation in astrocyte conditioned medium supplemented with 20 ng/ml BMP4 (mean±SEM, n = 3 independent experiments). No other neuronal phenotypes (i.e. Dopaminergic, Cholinergic) were detected. Scale bars = 10 µm. Abbreviations: BMP-4 = Bone morphogentic protein-4, GAD 65/67 = Glutamic acid decarboxylase 65/67, DARPP-32 = Dopamin and cAMP regulated phosphoprotein-32, Map-2 = Microtubule associated protein-2. (TIF) [file pone.0020941.s006.tif]

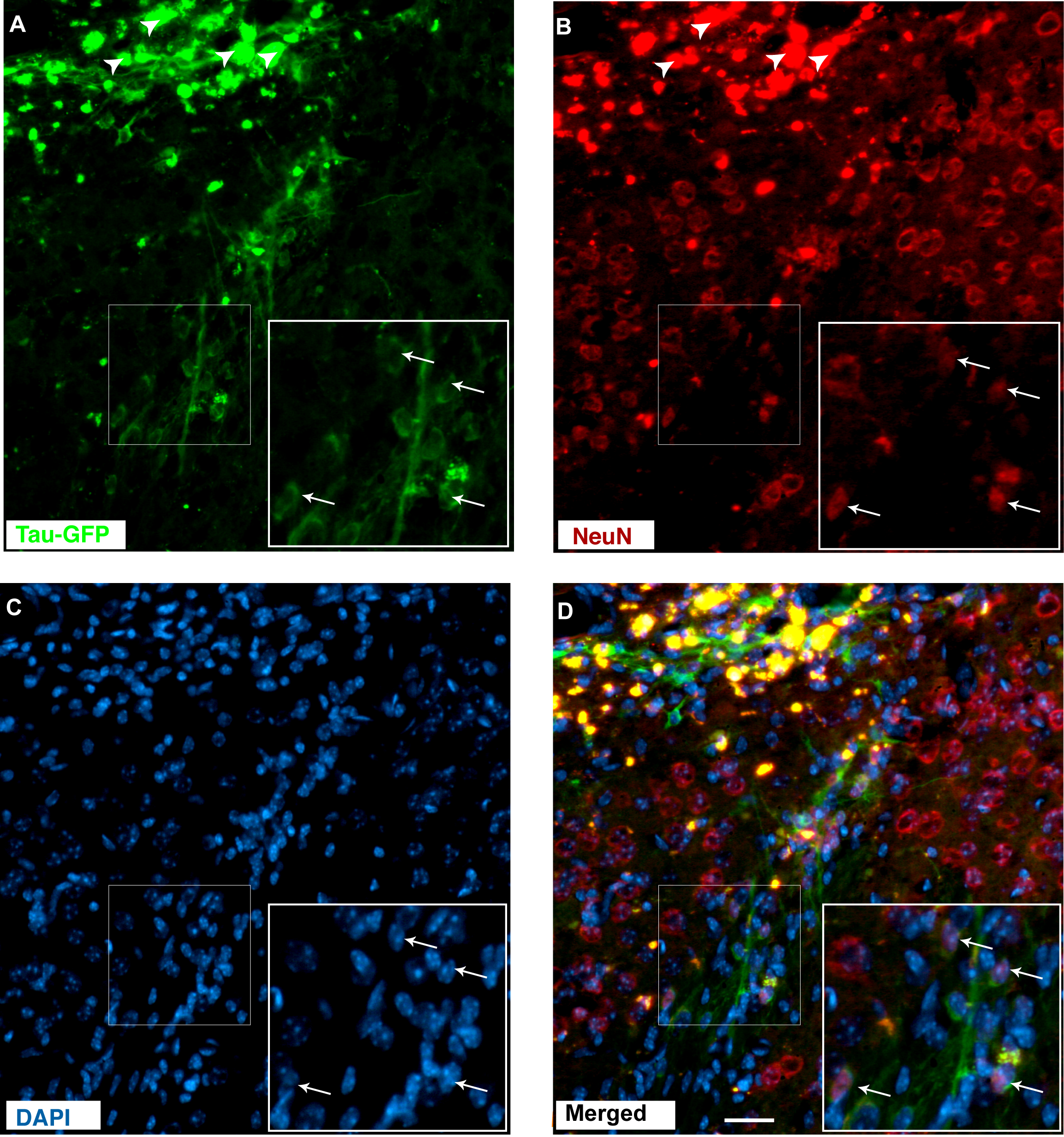

Supplement: Figure S7 — Sorted P1 cells implanted into the intact adult mouse striatum. Freshly sorted cells from P1 population were resuspended in NSC medium at a density of 5×10^4^ cells/µl and injected (2 µl /animal) into the right intact striatum of C57-BL6 mice. Cell survival and maturation was analysed 4 weeks later. (A–D): Representative micrographs from the transplant region demonstrated that the majority of the implanted cells (GFP+) (A) And approximately 1% of the implanted cells (arrows) survived, of which roughly 20% expressed NeuN (B–D), a mature neuronal marker (see figure 5F for quantitative data). Scale bar = 20 µm. (TIF) [file pone.0020941.s007.tif]

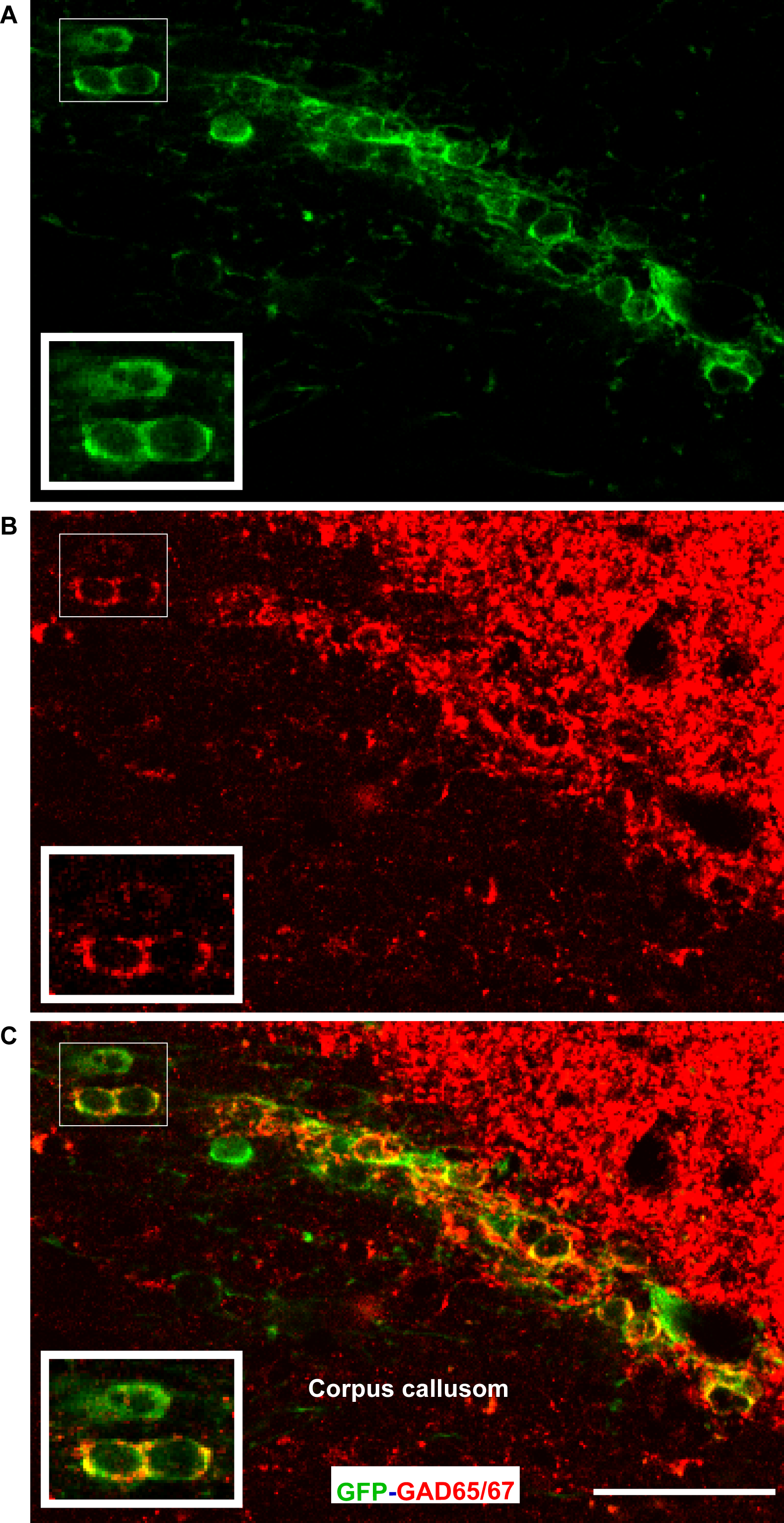

Supplement: Figure S8 — Sorted P1 cells give rise to GABAergic neurons after implantation into the adult mouse striatum. (A–C) Representative confocal micrographs illustrate that implanted P1 neuronal cells (A, green) that were treated with BMP4 survive and acquire GABAergic phenotype by expressing GAD65/67 (B; red and c, merged) 4 weeks post implantation. Scale bar = 20 µm. (TIF) [file pone.0020941.s008.tif]

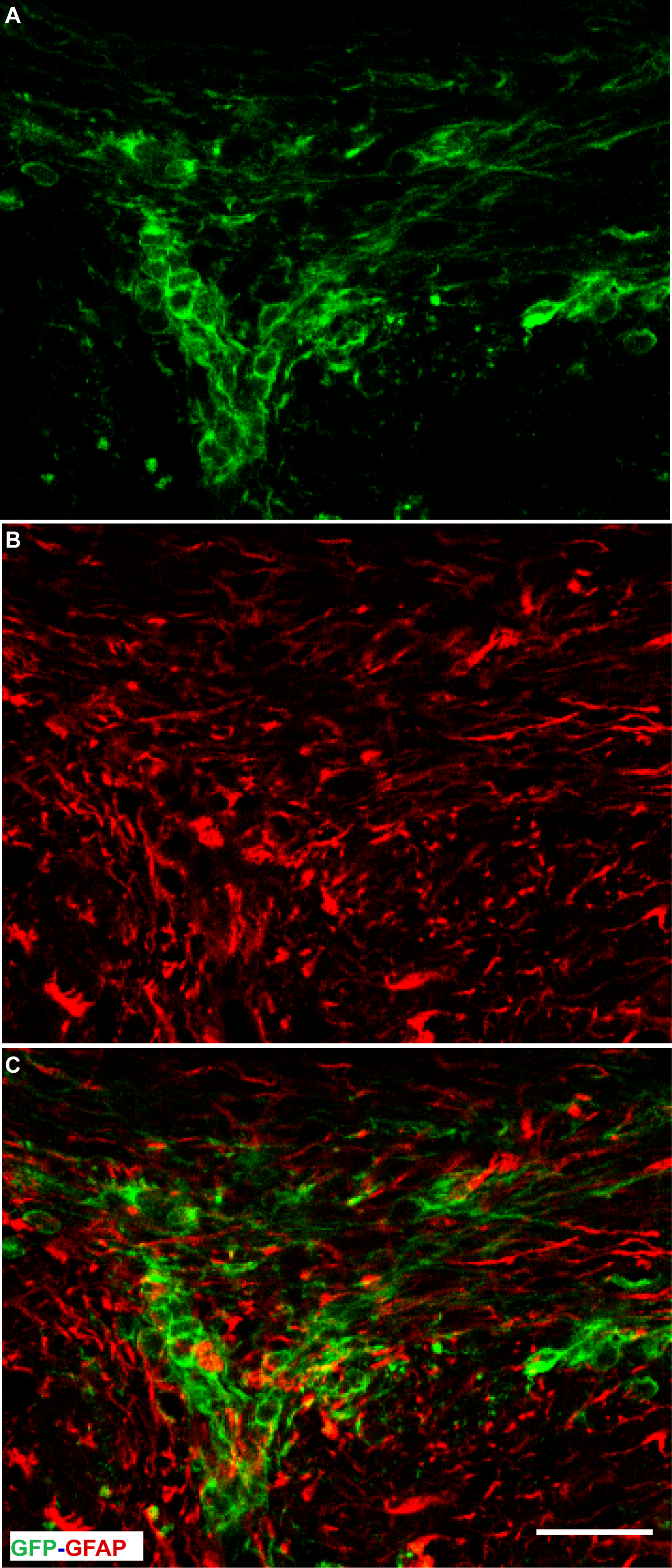

Supplement: Figure S9 — Sorted P1 cells do not differentiate into astrocytes after implantation into the adult mouse striatum. (A–C): Representative confocal micrographs show that implanted BMP4 treated P1 neuronal cells (A, green) survive but do not differentiate into GFAP expressing cells (B, red) as evident in the merged picture (C). Scale bar = 20 µm. (TIF) [file pone.0020941.s009.tif]
